# Targeting fibrinogen-like protein 1 enhances immunotherapy in hepatocellular carcinoma

**DOI:** 10.1172/JCI164528

**Published:** 2023-05-01

**Authors:** Mingen Lin, Jing He, Xinchao Zhang, Xue Sun, Wenjing Dong, Ruonan Zhang, Yanping Xu, Lei Lv

**Affiliations:** 1 Ministry of Education Key Laboratory of Metabolism and Molecular Medicine, Department of Biochemistry and Molecular Biology, School of Basic Medical Sciences, Fudan University, Shanghai, China.; 2 Tongji Hospital, Shanghai Key Laboratory of Signaling and Disease Research, Frontier Science Center for Stem Cell Research, School of Life Sciences and Technology, Tongji University, Shanghai, China.

**Keywords:** Cell Biology, Immunology, Cancer immunotherapy, Immunotherapy, Liver cancer

## Abstract

How cancer cells evade the therapeutic effects of immune checkpoint blockade is largely unknown. Here, we report that fibrinogen-like protein 1 (FGL1), a newly identified immune checkpoint ligand, was modified by acetylation at Lys 98 in hepatocellular carcinoma (HCC), which targeted it for proteasomal degradation. Sirtuin 2 (SIRT2) deacetylated and stabilized FGL1, thus promoting immune evasion. Notably, the SIRT2 inhibitor 2-Cyano-3-[5-(2,5-dichlorophenyl)-2-furanyl]-N-5-quinolinyl-2-propenamide (AGK2) enhanced acetylation of FGL1 and reduced FGL1 protein levels in vitro. The combination of AGK2 and programmed death ligand 1 (PD-L1) blockade effectively suppressed tumor growth and improved overall survival of mice. Furthermore, aspirin, an old drug, could directly acetylate FGL1 at Lys 98 and promote its degradation in vitro. Aspirin enhanced the immunotherapeutic efficacy, induced tumor regression, and extended the lifespan of tumor-bearing mice. Furthermore, the SIRT2/FGL1 axis was expressed in HCC specimens. Collectively, these findings unveil an acetylation-mediated regulation of FGL1, identify a potential target for HCC immunotherapy, and provide therapeutic strategies for the clinical treatment of HCC.

## Introduction

The use of checkpoint mechanisms to evade immune surveillance is one of the hallmarks of cancer (1, 2), but it is also a vulnerability. Antibodies targeting these negative regulators promote antitumor immunity and lead to improved clinical outcomes in a variety of cancers (3). Blockade of cytotoxic T lymphocyte–associated protein 4 (CTLA-4) by a specific antibody resulted in survival benefits for 20% of patients with melanoma, and blockade of the T cell checkpoint receptor or ligand, like programmed cell death 1 (PD-1) and programmed death ligand 1 (PD-L1), achieved responses as high as 30%–40% in several solid tumors (1). Immune checkpoint inhibitors (ICIs) are also the backbone of systemic therapies under development for the treatment of hepatocellular carcinoma (HCC). However, only 15%–20% of patients with HCC responded to ICI treatment and achieved objective remission and prolonged survival (4). Hence, the development of effective systemic therapies for HCC is an urgent unmet need in the clinical treatment of liver cancer. One promising strategy that may improve clinical outcomes for patients with HCC involves the targeting of another immune checkpoint in combination with current ICI therapies (1, 5).

Lymphocyte-activation gene 3 (LAG-3) is a transmembrane protein primarily found on activated T cells and functions as an inhibitory receptor (6, 7). Similar to PD-1, LAG-3 negatively regulates the proliferation, activation and homeostasis of both CD4^+^ and CD8^+^ T cells (8–13) and acts as a brake for CD8^+^ T cells in response to viral infections and tumors (14–17). Strikingly, a recent study revealed that fibrinogen-like protein 1 (FGL1) is a major functional ligand of LAG-3, independent of MHC class II (MHC-II) (18). The interaction between FGL1 and LAG-3 inhibited antigen-specific T cell activation, and, more important, ablation of FGL1 in mice promoted T cell immunity (18), indicating that FGL1 is an attractive target in cancer immunotherapy and potentially mediates the resistance to classic ICI therapies. Thus, it is also important to study the regulation of FGL1, which may provide strategies to enhance the efficacy of PD-1/PD-L1 blockade therapy and improve patient outcomes.

Posttranslational modifications (PTMs), including phosphorylation, ubiquitination, and acetylation, have been reported to provide proteins with precise regulation in both physiological and pathological processes (19). Lysine acetylation of nonhistone proteins is an evolutionarily conserved PTM occurring in prokaryotes and eukaryotes (20). Recently, accumulating evidence showed that acetylation enables proteins to control cellular signaling and biological events in a subtle and efficient manner, and the regulatory effects of acetylation can be attributed to altered catalytic activity, protein-protein interaction, subcellular localization or protein stability (21–25). Considering the importance of FGL1 in tumor immunity, it is worth determining whether FGL1 is acetylated and identifying the biological function of acetylation.

Here, we report that FGL1 could be acetylated at Lys 98, which led to its proteasomal degradation. Sirtuin 2 (SIRT2) deacetylates FGL1 and increased its protein stability. Notably, we show that aspirin could robustly increase the acetylation of FGL1 and promoted its degradation. Importantly, both pharmacological inhibition of SIRT2 and aspirin treatment sensitized HCC to anti–PD-L1 therapy. This study reveals a possible molecular mechanism for immune evasion of HCC and provides strategies for boosting the efficacy of anti–PD-1/anti–PD-L1 therapy.

## Results

### Acetylation at Lys 98 promotes FGL1 proteasomal degradation.

**Figure 1:**
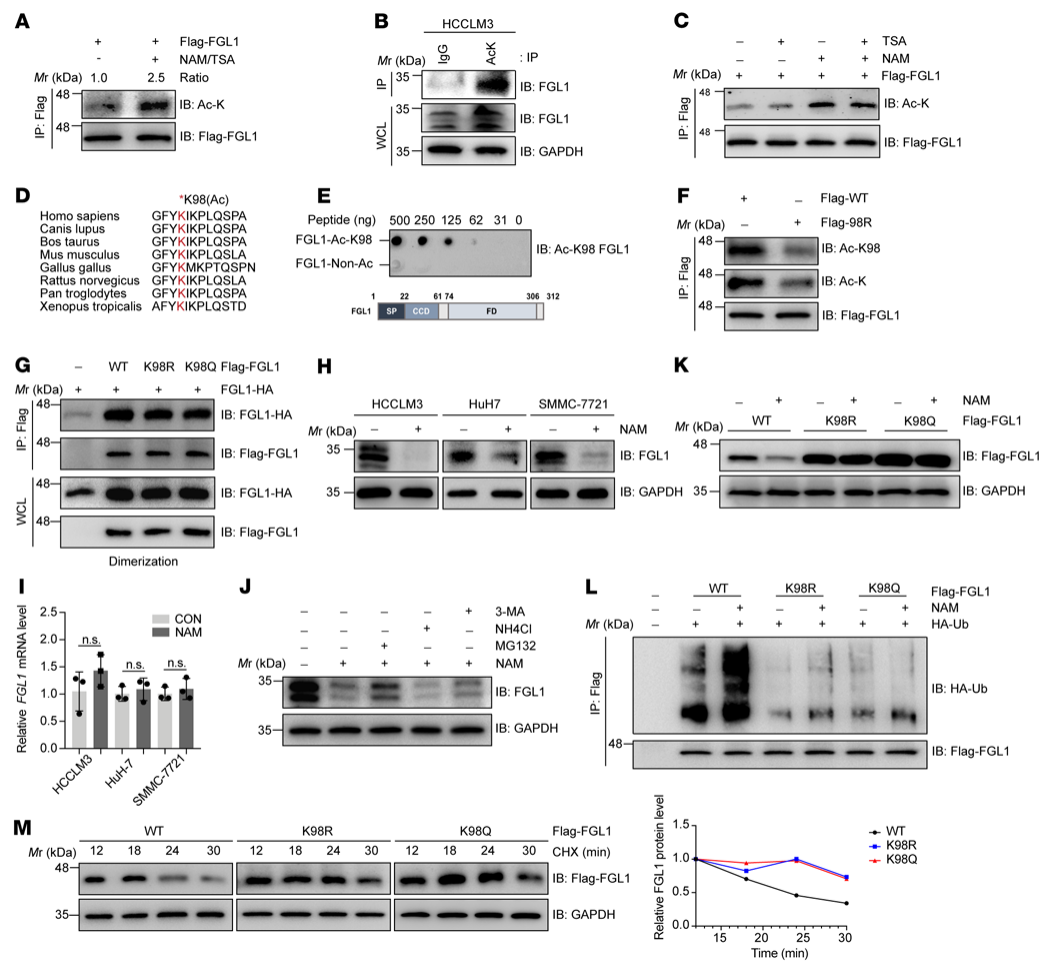
Acetylation at Lys 98 promotes FGL1 degradation via a ubiquitin-proteasome pathway. (**A**) Immunoblot (IB) analysis of Flag-FGL1 acetylation in HEK293T cells stably expressing Flag-FGL1 in the presence or absence of the deacetylase inhibitors TSA (5 μM, 16 h) and NAM (10 mM, 6 h). FGL1 acetylation was detected with an anti–acetylated lysine antibody. (**B**) IB analysis of whole-cell lysates (WCLs) and anti-lysine immunoprecipitates derived from HCCLM3 cells with IgG as a negative control. (**C**) IB analysis of acetylated, stably expressed Flag-FGL1 in HEK293T cells treated with TSA (5 μM, 16 h), NAM (10 mM, 6 h), or both. (**D**) Alignment of amino acid sequences of FGL1 containing Lys 98 across species. (**E**) The specificity of the site-specific anti–FGL1 acetylation antibody was determined by dot blot. (**F**) IB analysis of FGL1 acetylation derived from HEK293T cells transfected with WT Flag-FGL1 or the K98R mutant. FGL1–Non-Ac, nonacetylated FGL1. FD, fibrinogen domain; SP, signal peptide; CCD, the coil-coil domain. (**G**) IB analysis of WCLs and anti-Flag IPs derived from HEK293T cells transfected with the indicated constructs. (**H**) IB analysis of endogenous FGL1 in multiple HCC cell lines treated or not with NAM (10 mM, 6 h). (**I**) qPCR analysis of relative *FGL1* mRNA levels in multiple HCC cell lines treated or not with NAM. Data indicate the mean ± SD of 3 independent experiments. CON, control. (**J** IB analysis of endogenous FGL1 in HCCLM3 cells treated with NAM in the presence or absence of the proteasome inhibitor MG132, the lysosome inhibitor NH_4_Cl, or the autophagy inhibitor 3-MA. (**K**) IB analysis of FGL1 in HEK293T cells transfected with WT Flag-FGL1 or K98 mutant plasmids with or without NAM treatment. (**L**) WT Flag-FGL1 and the K98 mutants were cotransfected with HA-Ub into HEK293T cells with or without NAM treatment. The level of FGL1 ubiquitylation was determined by IB analysis. (**M**) IB analysis of turnover rates in HEK293T cells stably expressing WT Flag-FGL1 or the K98R or K98Q mutants treated with 100 μg/mL cycloheximide (CHX). *M*r, relative molecular mass; Ac-K, acetylated lysine.

The expression regulation and function of FGL1 have been studied comprehensively (18, 26–30), however, how FGL1 protein is regulated by PTMs remains elusive. Notably, FGL1 was identified as a potential target of acetylation modification by a mass spectrometry study in the PhosphoSitePlus database (31). To confirm the acetylation regulation of FGL1, we induced ectopic expression of Flag-tagged FGL1 in HEK293T cells and measured FGL1 acetylation levels using an anti–acetylated lysine antibody. We observed that the acetylation of FGL1 was enhanced approximately 2.5-fold after treatment with nicotinamide (NAM), an inhibitor of the SIRT family of deacetylases, and trichostatin A (TSA), an inhibitor of histone deacetylase (HDAC) I, II, and IV (Figure 1A). Consistently, acetylation of endogenous FGL1 was also detected (Figure 1B). Furthermore, we found that TSA treatment had no effect on FGL1 acetylation (Figure 1C and Supplemental Figure 1A; supplemental material available online with this article; https://doi.org/10.1172/JCI164528DS1), whereas NAM increased the acetylation of FGL1 in a dose- and time-dependent manner (Supplemental Figure 1, B and C). Interestingly, FGL2, another member of the FGL family, has also been reported to play a role in HCC progression and immune evasion (32, 33). We thus wondered whether FGL2 could be acetylated in HCC cells. Our results showed no detectable acetylation band of FGL2 in the endogenous IP with anti–acetylated lysine antibody (Supplemental Figure 1, D and E), indicating that acetylation may only occur on FGL1 in HCC cells. To determine the acetylation site of FGL1, we performed a screening via mutation of each FGL1 lysine (K) individually to arginine (R) and examined the acetylation levels of the mutants. Our results showed that mutation of Lys 98, but not other lysine residues, dramatically reduced the acetylation level of FGL1, demonstrating that Lys 98 is the major acetylation site of FGL1 (Supplemental Figure 1, F and G). Consistent with this finding, previous mass spectrometric data also indicated that Lys 98 of FGL1, which is highly conserved among species (Figure 1D), was a potential acetylation site. A site-specific antibody generated against acetyl-K98 of FGL1 (Figure 1E and Supplemental Figure 1H) recognized the acetylated WT FGL1, but not the K98R mutant (Figure 1F and Supplemental Figure 1I). Together, these results indicate that FGL1 was acetylated at Lys 98. It was reported that FGL1, like PD-L1, can potentially form a dimerization structure (34). We then verified whether acetylation of FGL1 regulates its dimerization and found that there was no detectable change in protein dimerization between WT FGL1 and the K98 mutants (Figure 1G).

Next, we determined the effect of NAM on the endogenous acetylation levels of FGL1. Surprisingly, instead of seeing increased acetylation levels, we observed that FGL1 protein levels were significantly decreased in multiple HCC cell lines treated with NAM (Figure 1H and Supplemental Figure 1J), while FGL1 mRNA levels were not changed (Figure 1I). Given that FGL1 is a liver-secreted protein (18), we examined whether acetylation of FGL1 decreases FGL1 levels by enhancing its secretion. The results showed that the secretion of FGL1 was reduced, and not increased, upon NAM treatment (Supplemental Figure 1K), implying that acetylation might affect the protein stability of FGL1. Of note, NAM-induced FGL1 downregulation could be partially rescued by MG132 treatment (a proteosome inhibitor), but not affected by NH_4_Cl (a lysosome inhibitor) or 3-methyladenine (3-MA) (an inhibitor of autophagy) (Figure 1J), suggesting that acetylation may promote FGL1 degradation via a ubiquitin-proteasome pathway. Interestingly, both acetylation-deficient (FGL1^K98R^) and acetylation-mimetic (FGL1^K98Q^) mutants were resistant to NAM treatment compared with WT FGL1 (Figure 1K and Supplemental Figure 1L). Moreover, inhibition of deacetylases with NAM increased the ubiquitination levels of WT FGL1, but not of the FGL1 mutants (Figure 1L), demonstrating that acetylation of FGL1 promoted its ubiquitination. Consistent with this, the turnover rate of WT FGL1 was much faster than that of the Lys 98 mutants (Figure 1M). Together, these results suggest that acetylation at Lys 98 promoted degradation of FGL1 via a ubiquitin-proteasome pathway.

### SIRT2 deacetylates and stabilizes FGL1 protein.

**Figure 2:**
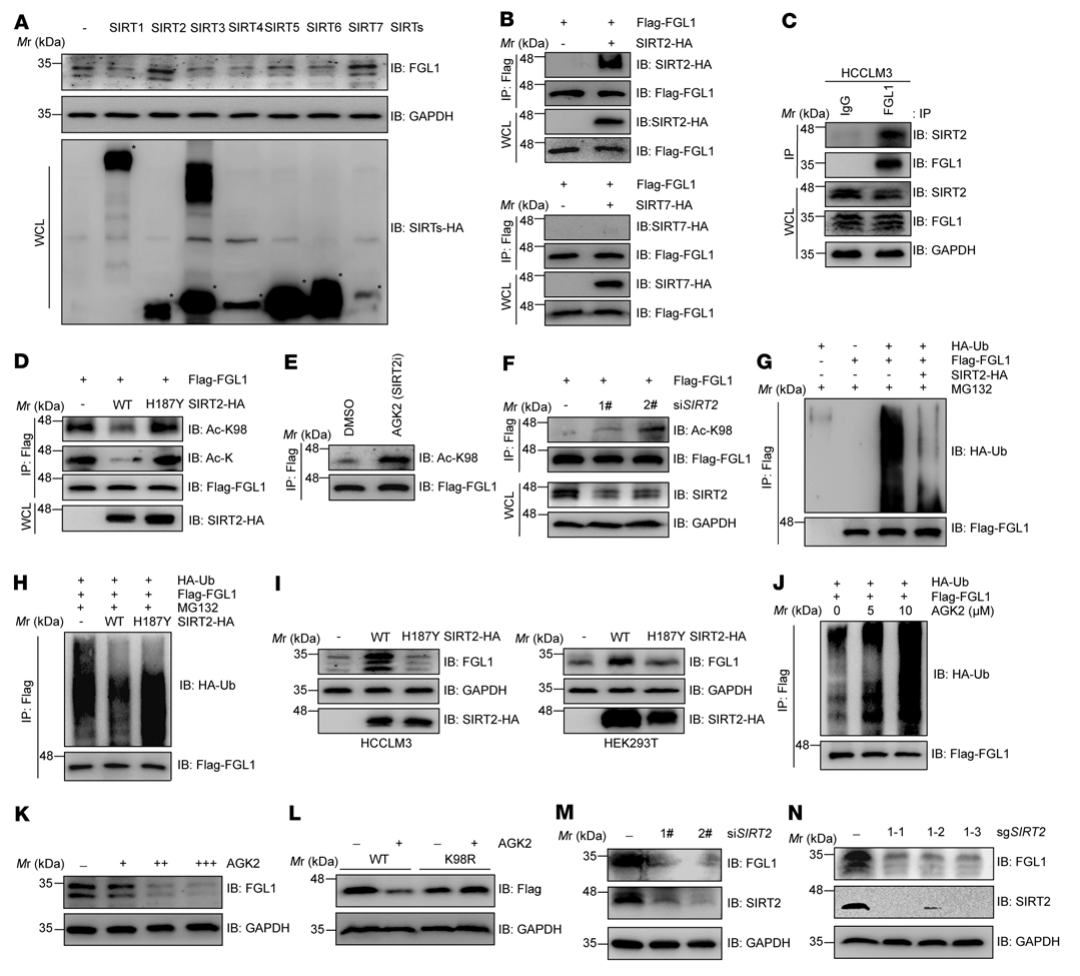
SIRT2 deacetylates and stabilizes FGL1. (**A**) IB analysis of endogenous FGL1 in HCCLM3 cells transfected with SIRTs. The interactions between FGL1 and SIRT2 or SIRT7 were determined by co-IP followed by IB analysis. (**B**) HEK293T cells were transfected with Flag-FGL1 and SIRT2-HA or SIRT7-HA as indicated. The interactions between FGL1 and SIRT2 or SIRT7 were determined by co-IP and IB analysis. (**C**) Endogenous interaction between FGL1 and SIRT2 in HCCLM3 cells was determined by IP and IB analysis. IgG was used as a negative control. (**D**) HEK293T cells stably expressing Flag-FGL1 were transfected with SIRT2-HA or its catalytic mutant as indicated. The acetylation of FGL1 was analyzed with pan– or site-specific anti–FGL1 acetylation antibodies. (**E**) HEK293T cells stably expressing Flag-FGL1 were treated or not with AGK2. Site-specific anti–FGL1 acetylation antibodies were used to determine the acetylation of FGL1 by IB analysis. (**F**) HEK293T cells stably expressing Flag-FGL1 were transfected with or without an siRNA oligonucleotide targeting *SIRT2*. The knockdown efficiency and the acetylation level of FGL1 were analyzed by IB. (**G**) IB analysis of FGL1 ubiquitination levels in HEK293T cells transfected with Flag-FGL1, SIRT2-HA, or HA-Ub. (**H**) IB analysis of FGL1 ubiquitination levels in HEK293T cells transfected with SIRT2-HA or its catalytic mutant. (**I**) IB analysis of both endogenous and exogenous FGL1 in HCCLM3 or HEK293T cells transfected with SIRT2-HA or its catalytic mutant. (**J**) IB analysis of FGL1 ubiquitination levels in HEK293T cells transfected with Flag-FGL1 and HA-Ub and treated with or without AGK2. (**K**) IB analysis of endogenous FGL1 in HCCLM3 cells treated with AGK2 (10–40 μM) for 8 hours. (**L**) IB analysis of FGL1 in HEK293T cells transfected with WT FGL1 or the K98R mutant under treatment with AGK2 for 8 hours. (**M**) IB analysis of endogenous FGL1 in WT and *SIRT2*-KO SMMC-7721 cells. (**N**) IB analysis of endogenous FGL1 in WT and *SIRT2*-KO HCCLM3 cells.

Previous results showed that NAM treatment led to a significant increase of FGL1 acetylation, whereas TSA treatment caused negligible changes in acetylation levels (Figure 1C and Supplemental Figure 1A), implying that FGL1 might be deacetylated by NAD^+^-dependent class III deacetylases. To find out which SIRT is responsible for the deacetylation of FGL1 at Lys 98, we induced overexpression of SIRTs in HCCLM3 and HEK293T cells and determined their effects on FGL1 protein levels. We found that both SIRT2 and SIRT7 increased FGL1 protein levels (Figure 2A and Supplemental Figure 2A), but only SIRT2 exhibited a physical interaction with FGL1 (Figure 2, B and C). Furthermore, coexpression of SIRT2, but not its catalytic inactive mutant H187Y, with FGL1 in HEK293T cells caused a remarkable decrease of FGL1 acetylation at Lys 98 (Figure 2D), where the use of 2-Cyano-3-[5-(2,5-dichlorophenyl)-2-furanyl]-N-5-quinolinyl-2-propenamide (AGK2), a selective SIRT2 inhibitor, or an siRNA targeting *SIRT2* markedly increased Lys 98 acetylation of FGL1 (Figure 2, E and F), indicating that SIRT2 is the deacetylase of FGL1. Consistent with the previous notion that acetylation promotes FGL1 degradation, SIRT2, but not its catalytic inactive H187Y mutant, decreased the ubiquitination of FGL1 (Figure 2, G and H) and increased its protein levels (Figure 2I and Supplemental Figure 2B), suggesting SIRT2 relies on its catalytic activity to stabilize FGL1 via altered ubiquitination. We also found that AGK2 treatment enhanced the ubiquitination of FGL1 in a dose-dependent manner (Figure 2J) and decreased FGL1 protein levels in multiple HCC cell lines, as measured by both immunoblotting and ELISA (Figure 2K and Supplemental Figure 2, C–E), while the FGL1^K98R^ mutant was resistant to AGK2-induced degradation (Figure 2L). In line with this, both knockdown and depletion of SIRT2 using an siRNA or CRISPR/Cas9 markedly downregulated FGL1 protein abundance (Figure 2, M and N, and Supplemental Figure 2F). Collectively, these data demonstrate that SIRT2 was responsible for the stabilization of FGL1 via Lys 98 deacetylation.

### AGK2 promotes T cell activation in vitro and the combination of AGK2 and PD-L1 blockade effectively suppresses tumor growth in vivo.

Since FGL1 is a newly identified immune checkpoint ligand (18) and inhibition of SIRT2 by AGK2 could decrease FGL1 levels in HCC cells, we then determined whether pretreatment of tumor cells with AGK2 would affect T cell activity. To this end, we established an in vitro coculture system (Supplemental Figure 3A) as previously described (35, 36). Jurkat cells were stimulated with phytohaemagglutinin (PHA) (Supplemental Figure 3B), which upregulated LAG-3 expression (Supplemental Figure 3C), and were then cocultured with 2 different HCC cells lines pretreated with AGK2. We found that the coculture with HCC cells decreased *IL2* mRNA levels in activated Jurkat cells, which could be rescued by AGK2 pretreatment (Supplemental Figure 3D). To further validate the role of AGK2 in the increased T cell activity, we performed a T cell–killing assay as previously described (37). We cocultured activated T cells (Supplemental Figure 3E) with HCC cells pretreated or not with AGK2. The results showed that AGK2 pretreatment significantly enhanced T cell–mediated killing of HCC cells (Supplemental Figure 3F). Overall, AGK2 promoted T cell activation and cytotoxic T lymphocyte (CTL) activity against HCC cells in vitro.

**Figure 3:**
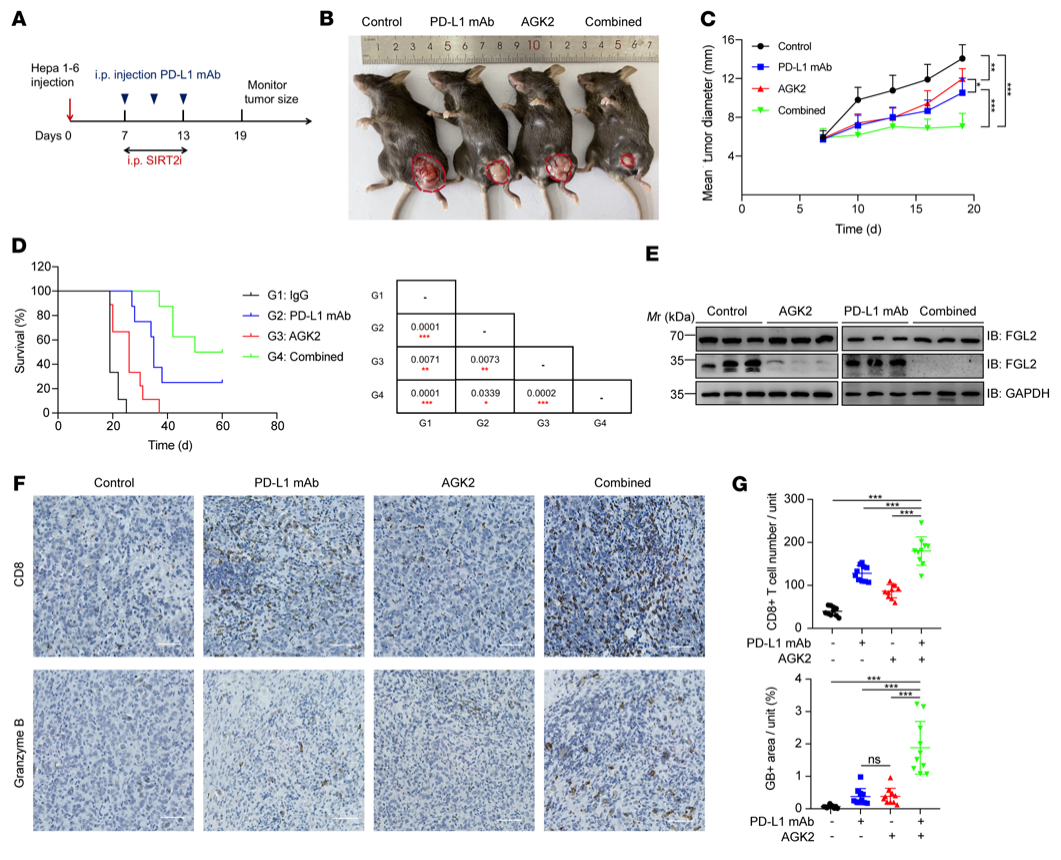
The combination of AGK2 and PD-L1 blockade effectively suppresses HCC tumor growth. (**A**) Schematic representation of the experimental procedure. (**B**) Representative tumors in C57BL/6 mice injected s.c. with Hepa 1-6 cells from different groups. (**C**) Tumor growth of Hepa 1-6 cells in C57BL/6 mice treated with control (black lines: *n* = 9), AGK2 (red lines: *n* = 9), anti–PD-L1 mAb (blue lines: *n* = 8), or their combination (green lines: *n* = 8). Tumor growth is shown as the mean tumor diameter ± SD. (**D**) Kaplan–Meier survival curves for each treatment group (black lines: *n* = 9, red lines: *n* = 9, blue lines: *n* = 8, green lines: *n* = 8). The *P* values were calculated using a 2-sided Gehan-Breslow-Wilcoxon test. (**E**) IB analysis of FGL1 and FGL2 protein levels in Hepa 1-6 tumor tissues from mice in each group, with GAPDH used as the internal control. (**F**) IHC of CD8^+^ T cell infiltration and granzyme B in Hepa 1-6 tumor tissues. Scale bars: 100 μm. Data represent the mean ± SD from 5 independent samples of each group. (**G**) Statistical differences for **F** were determined by Bonferroni-adjusted *P* values. *P < 0.05, **P < 0.01, and ***P < 0.001, with raw *P* values derived from an unpaired Student’s *t* test.

Both PD-L1 and FGL1 are immune-inhibitory checkpoints and function via different mechanisms. Therefore, blocking both pathways using anti–PD-L1 and anti-FGL1 antibodies dramatically improved the efficacy of immunotherapy (18). On the basis of our findings that inhibition of SIRT2 triggered degradation of FGL1 and enhanced T cell killing in vitro, we hypothesized that pharmacological inhibition of SIRT2 with AGK2 might also reduce FGL1 abundance and synergize with PD-L1 blockade to inhibit HCC growth in vivo. We used Hepa 1-6 and H22 syngeneic mouse models to assess the efficacy of anti–PD-L1 antibodies and/or AGK2 treatment (Figure 3A and Supplemental Figure 3G). AGK2 or anti–PD-L1 antibody treatment alone led to moderate inhibition of tumor growth and prolonged survival of mice (Figure 3, B and C, and Supplemental Figure 3H). Strikingly, the combination of AGK2 and anti–PD-L1 antibodies dramatically suppressed tumor growth, extended the survival of mice, and showed the best efficacy (Figure 3, C and D, and Supplemental Figure 3, H and I). Immunoblotting confirmed that i.p. injection of AGK2 decreased tumoral FGL1 levels in mice, whereas FGL2 protein levels were not affected by AGK2 treatment (Figure 3E). We next examined the level of tumor-infiltrating lymphocytes (TILs) using IHC staining and discovered that AGK2 treatment alone increased the tumor-infiltrating CD8^+^ T cell population and granzyme B levels, and, consistently, the combination of AGK2 and PD-L1 blockade had a significant, synergetic effect on the stimulation of tumor infiltration of CD8^+^ T cells and antitumor immunity (Figure 3, F and G, and Supplemental Figure 3, J and K). Of note, the effects of AGK2, as shown by the levels of granzyme B, appeared to be similar to the effects of PD-L1 blockade (Figure 3G). Together, these results indicated that pharmacological inhibition of SIRT2 might represent a strategy for enhancing the efficacy of PD-L1 blockade immunotherapy (Supplemental Figure 3L).

### Enforced acetylation of FGL1 by aspirin promotes FGL1 degradation.

**Figure 4:**
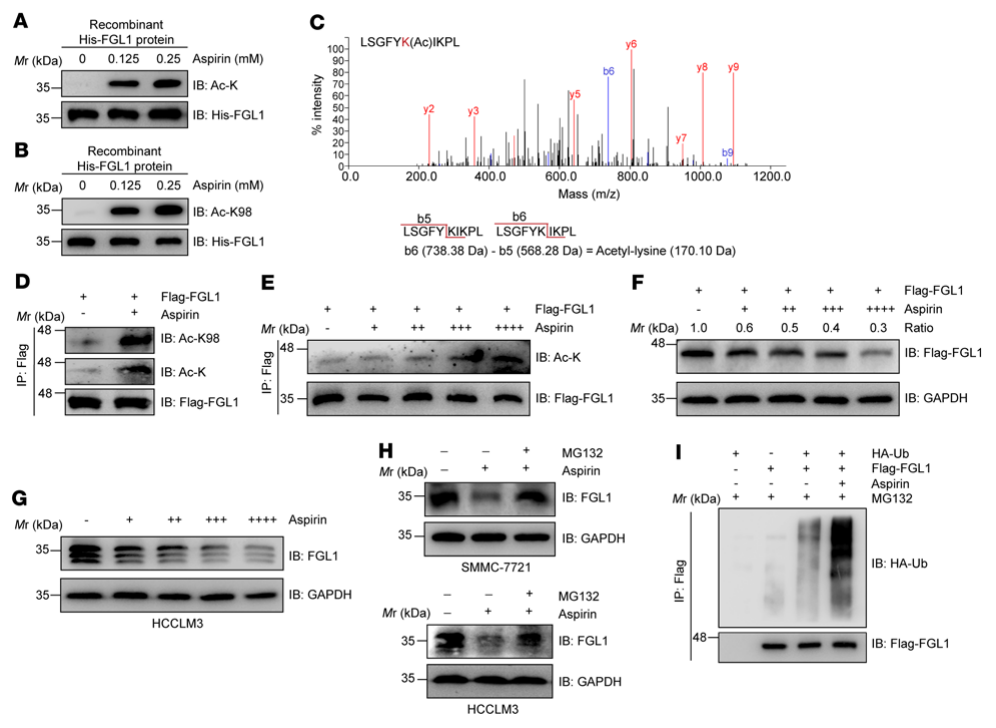
Aspirin acetylates FGL1 and promotes its degradation. (**A** and **B**) Recombinant His-FGL1 protein was treated or not with aspirin (0.125 mM or 0.25 mM) for 30 minutes in vitro. FGL1 acetylation was assessed by IB using a pan–anti–acetyl-lysine antibody or site-specific anti–FGL1 acetylation antibody. (**C**) Mass spectrometric detection of Lys 98 acetylation derived from recombinant His-FGL1 protein treated or not with aspirin (0.25 mM) for 30 minutes in vitro. The y ion peaks are shown in red, and the b ion peaks are shown in blue. The mass difference between b_6_ and b_5_ is the mass of the acetyl-lysine residue. (**D**) IB analysis of FGL1 acetylation levels in HEK293T cells stably expressing Flag-FGL1 and treated or not with aspirin (0.25 mM, 12 h). Pan– or site-specific anti–FGL1 acetylation antibodies were used as indicated. (**E**) IB analysis of FGL1 acetylation levels in HEK293T cells stably expressing Flag-FGL1 and treated with increasing concentrations of aspirin (+, 0.0625 mM; ++, 0.125 mM; +++, 0.25 mM; ++++, 0.5 mM) for 12 hours. (**F**) IB analysis of FGL1 protein levels in HEK293T cells stably expressing Flag-FGL1 and treated with increasing concentrations of aspirin (+, 0.0625 mM; ++, 0.125mM; +++, 0.25mM; ++++, 0.5 mM) for 24 hours. (**G**) IB analysis of endogenous FGL1 protein levels in HCCLM3 cells treated with increasing concentrations of aspirin (+, 0.0625 mM; ++, 0.125mM; +++, 0.25mM; ++++, 0.5mM) for 24 hours. (**H**) IB analysis of endogenous FGL1 protein levels in SMMC-7721 and HCCLM3 cells treated with aspirin (0.25 mM, 24 h) in the presence or absence of MG132 (10 μM, 6 h). (**I**) IB analysis of FGL1 ubiquitination levels in HEK293T cells stably expressing Flag-FGL1 and treated with 0.25 mM aspirin for 12 hours.

Aspirin was reported to be able to acetylate proteins, such as cyclic GMP-AMP synthase (cGAS), through direct transfer of an acetyl group (38). We then wondered whether aspirin could acetylate FGL1 at Lys 98 and promote the degradation of this protein. To test this hypothesis, recombinant FGL1 protein was incubated with increasing concentrations of aspirin in vitro. Impressively, we found that aspirin dramatically induced acetylation of FGL1 at Lys 98 using both pan- and site-specific acetylation antibodies (Figure 4, A and B, and Supplemental Figure 4, A and B), supporting the notion that aspirin can directly acetylate Lys 98 of FGL1. In line with this observation, we also detected acetylation of FGL1 at Lys 98 by mass spectrometry following aspirin treatment (Figure 4C). We next used aspirin to treat HCC cells with different concentrations for different durations. We found that aspirin treatment markedly augmented FGL1 acetylation in a dose-dependent manner, including at Lys 98 (Figure 4, D and E). Moreover, FGL1 protein levels were reduced in both 293T and HCC cell lines with aspirin treatment (Figure 4, F and G, and Supplemental Figure 4, C and D), which could be partially rescued by the proteasome inhibitor MG132 (Figure 4H), implying that aspirin induced the degradation of FGL1 via the ubiquitin-proteasome pathway. Consistently, we observed that aspirin treatment tremendously increased FGL1 ubiquitination (Figure 4I) and that the FGL1^K98R^ mutant was resistant to aspirin-induced degradation (Supplemental Figure 4E). These data suggest that aspirin induced FGL1 degradation by enhancing its acetylation at Lys 98. Considering that FGL1 is the major immune-inhibitory ligand of LAG-3 (18), we then assessed whether aspirin could reverse the “exhausted” T cell activity. To this end, PHA-stimulated Jurkat cells were cocultured with different HCC cells lines with or without aspirin pretreatment in a previously established in vitro coculture system (Supplemental Figure 4F). We found that the downregulation of *IL2* mRNA levels in activated Jurkat cells could be rescued with aspirin treatment (Supplemental Figure 4G). To further validate the role of aspirin in increased T cell activity, we performed the T cell–killing assay. We cocultured activated T cells with HCC cells pretreated or not with aspirin. Our results demonstrated that aspirin pretreatment enhanced T cell–mediated killing of HCC cells (Supplemental Figure 4H). Collectively, our data suggested that aspirin can increase the activity of CTLs against cancer cells.

### The combination of aspirin and PD-L1 blockade effectively suppresses tumor growth in vivo.

**Figure 5:**
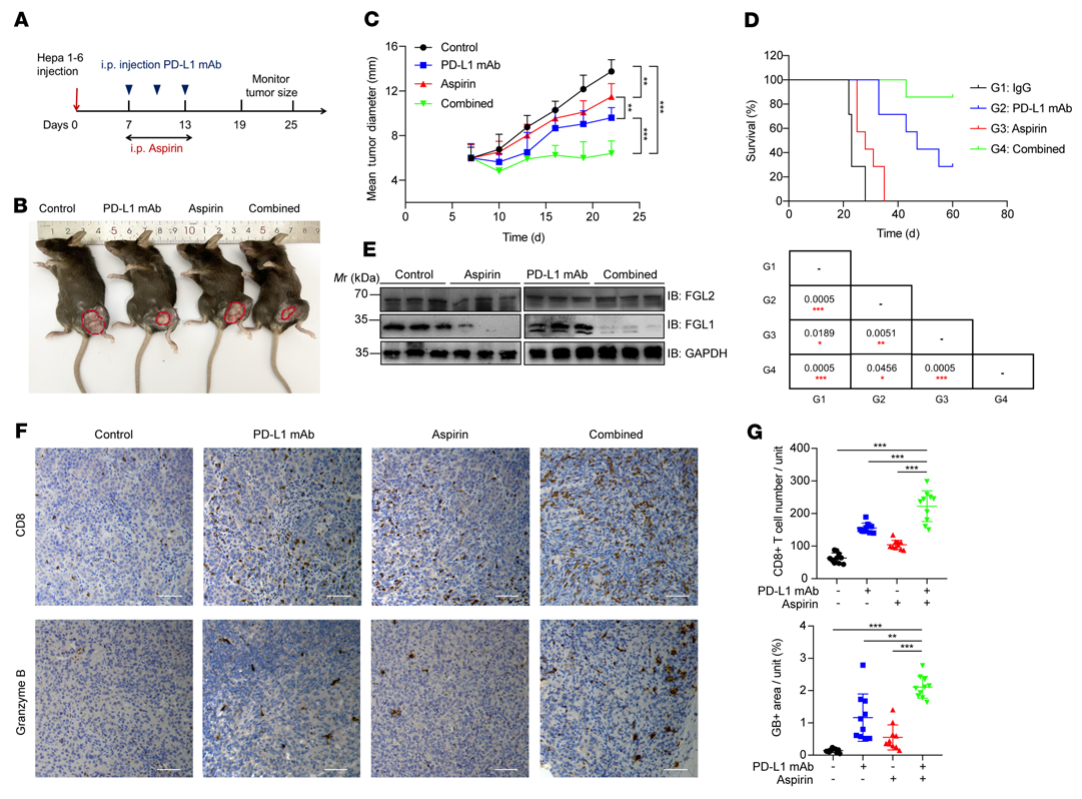
Aspirin downregulates FGL1 and enhances the efficacy of anti–PD-L1 immunotherapy. (**A**) Schematic representation of the experimental procedure. (**B**) Representative tumors in C57BL/6 mice injected s.c. with Hepa 1-6 cells from different groups with control, anti–PD-L1 mAb, aspirin, or combined treatment, respectively. (**C**) Tumor growth of Hepa 1-6 cells in C57BL/6 mice treated with control (black lines), aspirin (red lines), anti–PD-L1 mAb (blue lines), or their combination (green lines). *n* = 7 biologically independent animals per group. Tumor growth is shown as the mean tumor diameter ± SD. (**D**) Kaplan-Meier survival curves for each treatment group (G1, G2, G3, G4). *n* = 7 biologically independent animals per group. *p* values were calculated using a 2-sided Gehan-Breslow-Wilcoxon test. (**E**) IB analysis of FGL1 and FGL2 protein levels in Hepa 1-6 tumor tissues from each group with GAPDH as the internal control. (**F**) IHC analysis of CD8^+^ T cell infiltration and granzyme B expression in the Hepa 1-6 tumor mass as indicated. Scale bars: 100 μm. Data represent the mean ± SD of 5 independent samples from each group. (**G**) IHC results from **F** were quantified, and the statistical differences were determined by Bonferroni-adjusted *P* values. *P < 0.05, **P < 0.01, and ***P < 0.001, with raw *P* values derived from an unpaired Student’s *t* test.

To validate whether aspirin-induced FGL1 degradation benefits HCC immunotherapy in vivo, we selected PD-L1 blockade with aspirin for the combination therapy in Hepa 1-6 and H22 tumor models. Mice were treated with aspirin and/or anti–PD-L1 antibodies (Figure 5A and Supplemental Figure 5A). Intriguingly, the combination of aspirin and PD-L1 blockade resulted in a dramatic improvement in tumor suppression and long-term survival of mice compared with either agent alone (Figure 5, B–D, and Supplemental Figure 5, B and C). Decreased tumoral FGL1 levels in mice induced by aspirin treatment was validated by immunoblotting, while FGL2 protein levels were not affected by aspirin treatment (Figure 5E). Moreover, IHC staining of dissected tumors indicated that the CD8^+^ CTL population and activity were increased in the combined treatment group compared with other groups, implying that the superior therapeutic effect of combinatorial therapy was a result of increased numbers of intratumoral CD8^+^ T cells and enhanced cytotoxicity of those cells (Figure 5, F and G, and Supplemental Figure 5, D and E). Taken together, these results demonstrated that aspirin has the potential to enhance HCC immunotherapy.

Several anti–LAG-3 mAbs are in clinical trials and show promising anticancer activity (39). We thus compared the antitumor efficacy between FGL1 and LAG-3 inhibition both in vitro and in vivo. For the in vitro experiments, activated T cells were cocultured with HCC cells pretreated with or without AGK2 or aspirin or relatlimab, an anti–LAG-3 mAb (Supplemental Figure 5F). The results showed that both AGK2 treatment and aspirin pretreatment alone enhanced T cell–mediated cytotoxicity toward HCC cells better than LAG-3 blockade with relatlimab (Supplemental Figure 5G). For in vivo validation, AGK2 or aspirin treatment exhibited comparable efficacy with anti–LAG-3 mAb in suppressing tumor growth, and we detected no statistical significance between these 3 therapeutic strategies (Supplemental Figure 5, H and I). These findings suggest that blockade of FGL1 with AGK2 or aspirin is a promising strategy comparable to that of LAG-3 inhibition

### The levels of SIRT2 and relative acetyl-K98 FGL1 are negatively correlated and predict prognosis in HCC.

**Figure 6:**
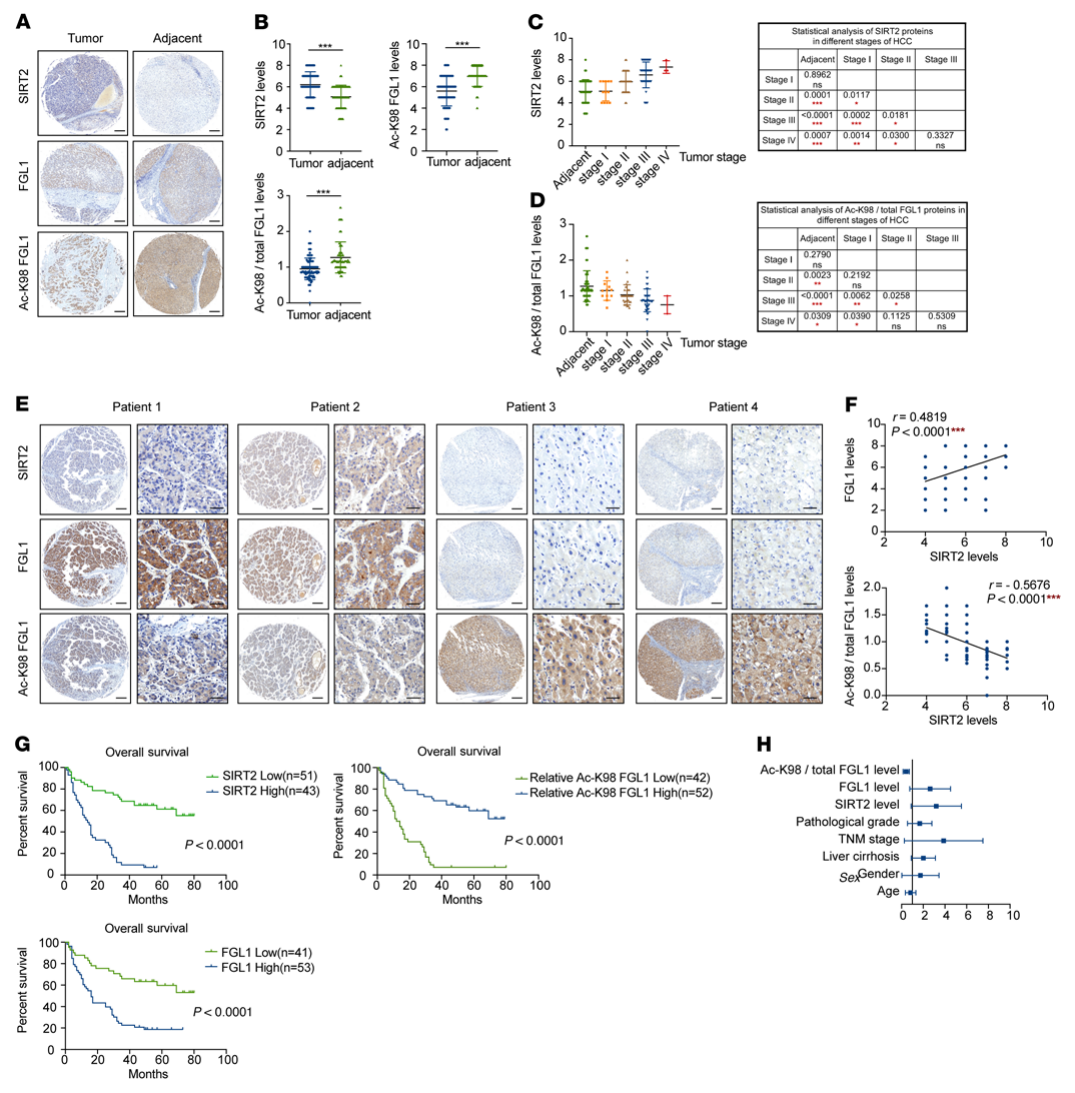
The levels of SIRT2 and relative acetyl-K98 FGL1 are negatively correlated and predict HCC prognosis. (**A** and **B**) Representative images of IHC staining for SIRT2, total FGL1 protein, and acetyl-K98 FGL1 in HCC and adjacent tissues (**A**). Scale bars: 200 μm. A total of 94 HCC cancer tissues and 86 adjacent tissues were analyzed. The IHC results were quantified and analyzed (**B**), and statistical differences were determined by Student’s *t* test. (**C** and **D**) Statistical analyses of SIRT2 and acetyl-K98/total FGL1 levels in HCC tumors of different stages. HCC tissues from 94 patients were categorized into different stages based on clinical data following AJCC classification. Statistical differences were determined by Student’s *t* test. *P < 0.05, **P < 0.01, and ***P < 0.001. (**E** and **F**) Representative images of IHC staining for SIRT2, total FGL1 protein, and acetyl-K98 FGL1 in HCC tissues derived from 4 patients (**E**). Scale bars: 200 μm (left panel) and 50 μm (right panel). (**F**) Statistical analysis of all samples from 86 paired samples. The IHC stains were scored, and Pearson’s correlation test was used to analyze the relationship between the levels of FGL1 and SIRT2 and the levels of acetyl-K98/total FGL1 and SIRT2. Certain dots on the graphs represent more than 1 specimen and are shown as overlapping. (**G**) Kaplan-Meier analysis of the correlation between SIRT2 protein levels and FGL1 and acetyl-K98/total FGL1 levels and overall survival of patients with HCC in the validated cohort (*n* = 94) with high and low expression of SIRT2, FGL1, and acetyl-K98/total FGL1. Cutoff values between groups with high and low expression were determined using the median value according to the total score obtained by combining the proportion and intensity scores. *P* values in **G** were calculated using the log-rank test. (**H**) Forest plot of multivariate analyses. See also Supplemental Table 7.

Our previous data showed that SIRT2 deacetylates and stabilizes FGL1, thus suppressing tumor immunity, suggesting that SIRT2 and acetylation of FGL1 may play an important role in the development of HCC. Notably, the mRNA levels of *SIRT2* are elevated in HCC and several other cancers compared with levels in normal tissues according to The Cancer Genome Atlas (TCGA) data (Supplemental Figure 6A). The findings that enhanced acetylation of FGL1 at Lys 98 by SIRT2 inhibition promoted the degradation of FGL1 prompted us to examine the correlation between Lys 98 acetylation and SIRT2 protein levels in HCC. We conducted IHC analyses on HCC samples from 94 patients, including a total of 86 paired samples that included adjacent liver tissues. Analysis of the IHC results revealed that SIRT2 protein levels were higher, whereas both the total and relative levels of acetyl-K98 FGL1 were lower in the HCC than in the adjacent tissues (Figure 6, A and B). The development of HCC can be divided on the basis of the clinical features into 4 stages (stages I, II, III, and IV), according to the American Joint Committee on Cancer (AJCC). To determine whether SIRT2 and relative acetyl-K98 FGL1 levels correlated with HCC progression, we analyzed the clinical data on tumor stages and IHC staining results. We found that SIRT2 protein levels were significantly increased in all tumor stages except stage I when compared with levels in adjacent tissues (Figure 6C and Supplemental Table 4). Moreover, there was a progressive increase in the levels of SIRT2 from stage I to stage II (*P* = 0.0117) and then to stage III (*P* = 0.0181, versus II, Figure 6C). These results are supported by the analysis of data derived from the University of Alabama at Birmingham Cancer data analysis portal (UALCAN) (https://ualcan.path.uab.edu/) (Supplemental Figure 6, B–D). Furthermore, SIRT2 protein levels correlated with tumor size (Supplemental Table 4). Taken together, these data suggest a possible role of SIRT2 in contributing to the progression, but not initiation, of HCC. Consistently, the levels of relative acetyl-K98 FGL1 were significantly decreased when compared with those in adjacent tissues with a pattern similar to that of SIRT2 (Figure 6D). Both FGL1 and relative acetyl-K98 FGL1 levels correlated with tumor size (Supplemental Tables 5 and 6). Furthermore, quantification of IHC staining of tumor tissues demonstrated that FGL1 protein levels were positively correlated with SIRT2 expression levels, while the levels of relative acetyl-K98 FGL1 were negatively correlated with SIRT2 expression levels (Figure 6, E and F). Together, these results provide clinical evidence for SIRT2-mediated deacetylation and stabilization of FGL1.

We further investigated the clinical relevance of SIRT2, FGL1, and FGL1–Lys 98 acetylation. Both SIRT2 and FGL1 expression levels were negatively correlated with a good HCC prognosis and lower expression of these 2 proteins was significantly associated with better overall survival, while relative acetyl-K98 FGL1 level predicted a good prognosis (Figure 6G). Notably, multivariate analysis demonstrated that high SIRT2 and FGL1 levels and low relative acetyl-K98 FGL1 levels were 3 independent predictors of overall survival in HCC (Figure 6H and Supplemental Table 7). These data indicate that SIRT2, FGL1, and relative acetyl-K98 acetylation may be potential biomarkers for HCC.

## Discussion

**Figure 7:**
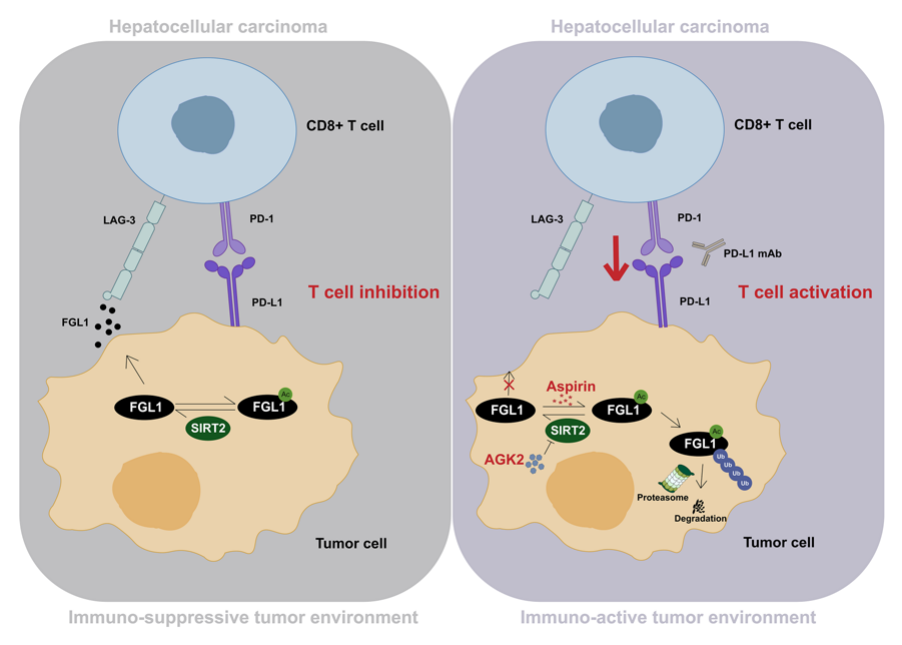
Inhibition of SIRT2 or aspirin treatment promotes cancer immunotherapy by inducing acetylation and proteasomal degradation of FGL1. Schematic diagram showing how the acetylation-dependent regulation of FGL1 enhances HCC immunotherapy. In HCC cells, SIRT2 deacetylates FGL1 at Lys 98 and increases its protein stability, thereby inhibiting T cell activation, leading to an immunosuppressive TME and evasion of immunosurveillance by tumor cells (left panel). Both pharmacological inhibition of SIRT2 by AGK2 and aspirin treatment increase Lys 98 acetylation of FGL1 and promote its proteasomal degradation, enhancing the efficacy of PD-L1 blockade–based immunotherapy.

Evasion of immune surveillance is a hallmark of cancer (40). Even though immunotherapies targeting classical checkpoints such as CTLA-4, PD-1, or PD-L1 have made substantial progress in different types of cancer, a large number of patients remain unresponsive to treatment, suggesting a vacancy for potential mechanisms of inhibitory ligand-receptor pathways (1). Recently, Wang and colleagues reported that FGL1 could negatively regulate immune responses by acting as a ligand of LAG-3 (18). Note that we observed an upregulation of *FGL1* mRNA levels in multiple types of cancer (Supplemental Figure 5), implying that the weak response of patients to immunotherapy may be due to the inhibitory tumor microenvironment (TME) (18). In this study, we revealed a mechanism of FGL1 regulation that affected its protein stability in HCC (Figure 7). We demonstrated that acetylation promoted FGL1 proteasomal degradation, whereas both FGL1^K98R^ and FGL1^K98Q^ mutants were resistant to acetylation-induced degradation. We found that SIRT2 was responsible for the deacetylation of FGL1 at Lys 98 and maintained the stability of FGL1, leading to an inhibitory TME. Notably, the acetylation level of FGL1 was reduced in HCC compared with levels in adjacent tissues. We demonstrated that decreased FGL1 acetylation in HCC may account for elevated FGL1 protein levels and an inhibitory TME. Importantly, AGK2, a selective inhibitor of SIRT2, remarkably increased Lys 98 acetylation of FGL1 and promoted its degradation. Given that deubiquitinase and ubiquitinase reversely regulate protein stability, it would be interesting in future studies to determine the underlying mechanism for how acetylation affects the stability of FGL1. Besides acetylation, other PTMs are also involved in the regulation of protein levels. PD-L1, a classical inhibitory ligand, has been reported to be glycosylated and ubiquitinated, and abnormal glycosylation contributes to its degradation by ER-associated protein degradation (37). Intriguingly, this process can be triggered by d-mannose treatment (41). Here, we have determined that FGL1 is regulated by acetylation, and it would be worth investigating how FGL1 is regulated by other PTMs and the possible interplay of these different PTMs.

Previous research has reported that aspirin could directly acetylate cGAS both in vitro and in vivo in an acetylase-independent manner (38). With recombinant FGL1 protein, we discovered that Lys 98 of FGL1 was dramatically acetylated by aspirin in vitro. Moreover, we found that aspirin directly acetylated FGL1 and induced its ubiquitination-mediated degradation. Furthermore, our data consistently showed that aspirin promoted FGL1 degradation in various human HCC cell lines. Recent research has consistently shown that aspirin use is associated with a time- and dose-dependent reduction in HCC risk (42). Moreover, the cumulative incidence of HCC- and liver-related mortality has been found to be significantly reduced with aspirin treatment (43). Importantly, the effective dose of aspirin is tolerated, safe, and feasible for clinical treatment of patients with HCC (38, 44–48). Note that the efficacy of aspirin treatment may be context associated because of the additional targets in the organism, and other potential targets and functions of aspirin need to be explored and studied.

CD8^+^ cytotoxic T cells play an important role in tumor regression. However, the immune responses are often impaired by an inhibitory ligand-receptor interaction, resulting in tumor development. In this study, our data demonstrate that AGK2 and aspirin could reverse the exhausted phenotype of CD8^+^ T cells. As shown in Figure 1K and Figure 4G, enhancement of FGL1 acetylation with AGK2 or aspirin decreased FGL1 protein levels, which promoted CD8^+^ cytotoxic T cell infiltration and granzyme B expression in the TME. Therefore, the combination of AGK2 or aspirin with anti–PD-1/anti–PD-L1 not only blocked FGL1/LAG-3 signaling but also abolished the PD1/PD-L1 pathway, thus boosting the efficacy of the immunotherapy. It is conceivable that pharmacological inhibition of SIRT2 may reprogram cancer cells to augment HCC immunotherapy.

Overexpression of SIRT2 in primary HCC is associated with increased microscopic vascular invasion and poor prognosis (49). In this study, we observed that SIRT2 was markedly increased in HCC compared with adjacent tissues. Consistently, FGL1 Lys 98 acetylation was markedly decreased in HCC. The clinical significance of SIRT2-mediated deacetylation and stabilization of FGL was further evidenced by the negative correlation between Lys 98 acetylation and SIRT2 expression in human HCC samples and the correlation between these molecular markers and the overall survival of patients with HCC. Our findings also suggest that low SIRT2 expression or high relative acetyl-K98 FGL1 levels may predict a better response to PD-1/PD-L1 blockade. In summary, our findings not only unveiled an acetylation-dependent regulation of FGL1 that may be utilized by tumors for immune evasion, but also linked SIRT2 and aspirin to HCC immunotherapy. Clinically, we provide a potential therapeutic target, strategies, and drug, and identify predictive markers for therapeutic efficacy and prognostic markers for patients with HCC.

## Methods

### Study design.

The object of this research was to investigate the mechanism and role of SIRT2 in mediating tumor immune evasion in HCC, as well as to evaluate combined therapy targeting SIRT2 or FGL1 deacetylation. Each biochemical experiment was replicated at least twice with similar results. Immunoblotting, IP, and quantitative PCR (qPCR) analyses, as well as in vivo ubiquitination assays and other assays were used to study the mechanism of SIRT2-mediated deacetylation and aspirin-mediated acetylation of FGL1. IHC, qPCR analysis, T cell–mediated tumor cell–killing assays, and other assays were conducted to analyze the activation of Jurkat cells, CD8^+^ T cell infiltration and function, as well as the correlation of protein expression in tumor samples from patients with HCC or from HCC mouse models. Blood from healthy volunteers was used to isolate human PBMCs. A human hepatocellular carcinoma tumor tissue microarray was acquired from Shanghai Outdo Biotech, with informed consent from all patients. For in vitro assays, at least 3 independent replicate experiments were performed. The investigators were blinded to the allocation of the groups for IHC staining. All other experiments were performed in a nonblinded manner. For in vivo studies, the sample sizes were in accordance with previously established models (18). Mice were randomly divided into different groups before treatments and monitored every 3 days after tumor implantation. Mice were humanely sacrificed at defined study endpoints.

### Cell culture, transfection, virus infection, and treatments.

HEK293T cells were a gift from the Molecular and Cell Biology Laboratory (Fudan University, Shanghai, China). HCCLM3, SMMC-7721 and Huh7 cell lines were provided by Zhuoyang Fan (Zhongshan Hospital, Fudan University, Shanghai, China). Hepa 1-6 cells were purchased from the Cell Bank at the Institute of Biological Sciences (Chinese Academy of Sciences, Shanghai, China). H22 cells were provided by Shu Zhang (Zhongshan Hospital, Fudan University, Shanghai, China). HEK293T, HCCLM3, SMMC-7721, Huh7, Hepa 1-6, and H22 cells were cultured in DMEM (Meilun Biotechnology). All media contained 10% FBS and 1% penicillin/streptomycin (P/S). Transfection was performed using EZ-trans (Life-iLab). For lentiviral infection, HEK293T cells were transfected with packaging vectors and core plasmids (pLenti-CRISPRV2 vector). The virus particles were then collected for infection. The target cells were incubated in lentivirus-containing medium with polybrene (10 μg/mL) for 48 hours and then subjected to puromycin selection. For deacetylase inhibitor treatment, TSA (5 μM, final concentration) or NAM (10 mM, final concentration) was added to the culture medium for 16 hours and 6 hours, respectively, before harvesting. AGK2 (TargetMol) or aspirin (Ouhe Technology) was added to the complete medium at the indicated concentrations and for the indicated durations.

### Plasmids.

Human cDNAs of protein SIRT1–7, ubquitin (Ub), and FGL1 were cloned into Flag-, Myc-, or HA-tagged destination vectors according to different experimental needs. Site-directed mutagenesis was performed using PCR with KOD Fx (TOYOBO). FGL1 plasmids were amplified, and final products were digested with DpnI enzyme (Takara) and then transformed into NcmDH5-α (NCM Biotech) for amplification. For the generation of CRISPR-KO cell lines, sgRNAs were subcloned into the pLenti-CRISPRV2 vector. The sgRNA sequences for human *SIRT2* KO were as follows: 5′-TCCGGTCAGTGGACTTCCTG-3; 5′-AGCGTTCGCTCTGCATGTAC-3; 5′-AGGTCGCAGAGTCATCTGTT-3.

### qPCR analysis.

Total RNA was isolated using the EZ-press RNA Purification Kit (EZ Bioscience). cDNA was synthesized using 4× Reverse Transcription Master Mix (EZ Bioscience) according to the manufacturer’s instructions. qPCR was performed with 2× SYBR qPCR Mix (KTSM, AlpaLife) using an Applied Biosystems 7300 Plus Sequence Detection System. All primers were synthesized by BioSune. Sequence information on the primers used is provided in Supplemental Table 1.

### Immunoblot and IP analysis.

Cells were lysed in NP-40 lysis buffer (150 mM NaCl and 50 mM Tris-HCl, pH 7.5) containing a protease inhibitor cocktail (ApexBio). Proteins were separated by SDS–PAGE and transferred onto nitrocellulose filter membranes. The membranes were incubated with the following antibodies: anti–acetylated lysine (9441, 1:500; Cell signaling Technology); anti-FGL1 (16000-1-AP, 1:1,000; Proteintech), anti-FGL2 (67152-1-Ig, 1:1,000; Proteintech); anti-GAPDH (60004-1-Ig, 1:3,000; Proteintech); anti-SIRT2 (ab211033, 1:1,000; Abcam); anti–LAG-3 (16616-1-AP, 1:1,000; Proteintech); anti-His (R1207-2, 1:1,000; HUABIO); anti-Flag (HOA012FL01, 1:5,000; AbHO); and anti-hemagglutinin (anti-HA) (HOA012HA01, 1:5,000; AbHO). The anti–acetyl-K98 FGL1 (acetyl-Lys 98-FGL1) antibody (generated by ABclonal Technology Biotech) was used at a dilution of 1:1,000. For secondary antibodies, sn HRP-conjugated anti–mouse secondary antibody (L3032, Signalway Antibody [SAB]) or an anti–rabbit secondary antibody (L3012, SAB) was used at dilutions of 1:3,000 and 1:1,000, respectively. For Flag-IP, lysates were collected and centrifuged (13,400 *g*, 15 min, 4°C). The supernatants were incubated with anti-Flag beads (HOA032FL01, AbHO) overnight at 4°C. Immune complexes were washed 5 times with NP-40 buffer and then subjected to SDS-PAGE. For endogenous IP, cells were lysed in NP-40 lysis buffer supplemented with a protease inhibitor cocktail, 5 μM TSA, and 10 mM NAM, followed by centrifugation (13,400 *g*, 15 min, 4°C). The supernatants were immunoprecipitated with anti-FGL1 antibody overnight and then incubated with Protein G Sepharose (17-0618-01, GE Healthcare) for 6 hours at 4°C. The immunoprecipitates were washed 5 times with NP-40 lysis buffer and boiled in 1× SDS loading buffer for immunoblot analysis. All immunoblot images were taken with a Tanon 5200 imaging system. The commercial antibodies used are listed in Supplemental Table 2.

### Trichloroacetic acid precipitation.

Trichloroacetic acid (TCA) precipitation was performed with the TCA Protein Precipitation Kit (Sangon Biotech) to concentrate the secreted FGL1 protein from the cell culture medium according to the manufacturer’s protocol. The samples were then subjected to SDS-PAGE.

### ELISA.

HCCLM3 and SMMC-7721 cells were pretreated with AGK2 (20 μM, 8 h) or aspirin (0.25 mM or 0.5 mM, 24 h). Soluble levels of FGL1 in cells from the different experimental groups were determined using the Human FGL1 ELISA kit (CUSABIO Technology, https://www.cusabio.com/) according to the manufacturer’s instructions.

### In vivo ubiquitination assay.

Thirty-six hours after transfection, cells were lysed in 1% SDS buffer (pH 7.5 Tris, 0.5 mM EDTA, 1 mM DTT) containing protease inhibitors and boiled for 10 minutes. Cell lysates were diluted 10-fold with 0.3% NP40 buffer and subjected to IP. The ubiquitination level was analyzed by immunoblotting.

### RNA interference.

SIRT2 knockdown was carried out using synthetic siRNA oligonucleotides purchased from RiboBio. Two effective target sequences were used to exclude off-target effects. Transfection of siRNAs was performed using Lipofectamine 2000 (Invitrogen, Thermo Fisher Scientific) following the manufacturer’s instructions. The knockdown efficiency was verified by immunoblotting. The following effective sequences were used: *SIRT2* siRNA no. 1, 5′-GAGGCCAUCUUUGAGAUCA-3′ and *SIRT2* siRNA no. 2 , 5′-AUGACAACCUAGAGAAGUA-3′

### Coculture experiments.

HCCLM3 and SMMC-7721 cells were pretreated with AGK2 or aspirin for the indicated duration, and then cancer cells were allowed to adhere to the plates overnight. Jurkat cells were activated with PHA (500 ng/mL) for 72 hours and then cocultured with pretreated HCCLM3 or SMMC-7721 cells at a ratio of 10:1 for 48 hours. After coculturing, the Jurkat cells were collected and subjected to qPCR analysis.

### T cell–mediated tumor cell–killing assay.

Human PBMCs collected from healthy donors were isolated from the blood. To acquire activated T cells, PBMCs were cultured in ImmunoCult-XF T cell expansion medium (10981, STEMCELL Technologies) with ImmunoCult Human CD3/CD28/CD2 T cell activators (25 μL/mL, 10970, STEMCELL Technologies) for the first 3 days and with IL-2 (10 ng/mL, Pepro Tech) for 1 week according to the manufacturer’s protocol. HCCLM3 cells were pretreated with AGK2 or aspirin for the indicated duration, and then tumor cells were allowed to adhere to the plates overnight. After that, the activated T cells were cocultured with the tumor cells at a ratio of 1:10 in DMEM/F12 medium containing anti-CD3 antibody (100 ng/mL, 160027, eBioscience, Thermo Fisher Scientific) and IL-2 (10 ng/mL) for 48 hours. Living tumor cells were washed with PBS, stained with 0.1% crystal violet, and then eluted with 33% (v/v) acetic acid followed by quantification using a spectrometer at an OD of 570 nm.

In experiments comparing the efficacy of FGL1 and LAG-3 inhibition, activated T cells were treated with the anti–LAG-3 mAb relatlimab (10 μg/mL, A2029, Selleck Chemicals) or control IgG (10 μg/mL, HG4K, Sino Biological) for 48 hours and cocultured with tumor cells.

### Mass spectrometric analysis.

To identify the acetylation sites of FGL1 after aspirin treatment in vitro, mass spectrometric analysis of digested peptides from different groups (DMSO or aspirin) was carried out. In brief, desalted peptides were analyzed using a TIMS-TOF Pro (Bruker Daltonics). TIMS-TOF Pro raw MS data were searched against a downloaded UniProt database (downloaded on December 2, 2021, containing 20,375 protein sequence entries) with the taxonomy of *Homo sapiens* and subjected to PEAKS Online Xpro Software analysis (version 1.6) for the identification of peptides and proteins.

### Animal experiments.

Female 6- to 8-week-old C57BL/6 mice and female 6- to 8-week-old BALB/c mice were purchased from JieSiJie Laboratory Animals and Qizhenlab, respectively. The syngeneic Hepa 1-6 cancer model was established by subcutaneously injecting 1 × 10^6^ Hepa 1-6 cells into the right flank of C57BL/6 mice. To establish an H22 syngeneic cancer model, 1 × 10^6^ of H22 murine hepatocarcinoma tumor cells were injected into the right flank of BALB/c mice. Tumor growth was monitored by electronic caliper every 3 days and is shown as the mean tumor diameter: (length + width)/2 (ref. 18). C57BL/6 mice were randomly divided on day 7, and BALB/c mice were randomly divided on day 4 after injection and subsequently treated i.p. with 100 μg anti–PD-L1 mAbs on days 7, 10, and 13 with IgG as a control. For AGK2 or aspirin treatment, mice were treated i.p. with 10 mg/kg AGK2 three times a week or with 50 mg/kg aspirin once a day. For survival studies, animals were sacrificed when the tumor volume exceeded 1,500 mm^3^ or when the tumor became ulcerated, with the ulcer diameter reaching 1.5 cm. The commercial antibodies used for the in vivo experiments are shown in Supplemental Table 2.

### IHC analysis.

Mouse Hepa 1-6 and H22 tumor were fixed with 4% paraformaldehyde (Beyotime) and then transferred to 70% ethanol. IHC analysis was performed as previously described (50). In brief, paraffin-embedded sections were deparaffinized with deparaffinization buffer (Solarbio Life Science). Antigen retrieval and elimination of endogenous peroxidase activity were achieved with 10 mM citric acid buffer (pH 6.0) and 3% H_2_O_2_, respectively, followed by blocking and antibody incubation. Detection and visualization procedures were performed following the manufacturer’s protocol. The commercial antibodies used for IHC staining are listed in Supplemental Table 3.

### Human tissue microarray analysis.

For human hepatocellular carcinoma tumor tissue microarray analysis, a paraffin HCC tissue microarray was acquired from Shanghai Outdo Biotech Co. Ltd with obtained informed consent from the patients. All procedures related to human subjects were approved by the Ethics Committee of Shanghai Outdo Biotech Company and the research was conducted according to the Declaration of Helsinki. IHC was performed according to the protocol described above. To quantify the IHC results, immunoreactivity was evaluated in accordance with intensity and area as previously defined (51). In brief, the percentage of immunoreactivity was determined on a scale from 0–5 (0 if 0% of the tumor cells showed positive staining, 1 if 0%–1% of cells were stained, 2 if 2%–10% stained, 3 if 11%–30% stained, 4 if 31%–70% stained and 5 if 71%–100% stained). The staining intensity was graded on a scale from 0 to 3 (0, no staining; 1, weak staining; 2, moderate staining; 3, strong staining). A total score was obtained combining the proportion and intensity scores (range: 0, 2–8). Statistical significance was set at *P* < 0.05. The commercial antibodies used for IHC staining are shown in Supplemental Table 3.

### Statistics.

All quantitative data are presented as the mean ± SD. Statistical analysis was performed using GraphPad Prism 8 (GraphPad Software). Data in the bar graphs represent the fold change or the percentage relative to control with the SD of 3 independent experiments. Student’s *t* test was performed to compare 2 groups of independent samples, and multiple comparisons were corrected using Bonferroni’s method. The Gehan-Breslow-Wilcoxon test was used to evaluate statistical differences between groups in the survival studies. A *P* value of less than 0.05 was considered statistically significant.

### Study approval.

All animal experiments and the procedures related to humans in this study were approved by the Ethics Committee of the Basic Medical School at Fudan University and the Ethics Committee of Shanghai Outdo Biotech Company, and all studies were conducted in accordance with Declaration of Helsinki principles. All animal experiments were executed in accordance with the ethical obligations approved by the department of laboratory animal science of Fudan University. Human blood samples were obtained from healthy volunteers, who gave informed consent.

## Author contributions

ML played major roles in designing and performing the experiments, analyzing the results, and organizing the figures. JH, XZ, XS, WD, and RZ helped conduct the experiments. YX and LL conceived and designed the study. LL supervised the study. ML and LL wrote the manuscript.

## Supplementary Material

Supplemental data

## Version 1 05/01/2023

Electronic publication
