# Supplementary material for: Targeting fibrinogen-like protein 1 enhances immunotherapy in hepatocellular carcinoma
Source: J Clin Invest. 2023 May 1;133(9):e164528. doi: 10.1172/JCI164528 (PMC10145938; doi:10.1172/JCI164528)
Supplement: Supplemental data [file jci-133-164528-s019.pdf]

Supplemental Figures

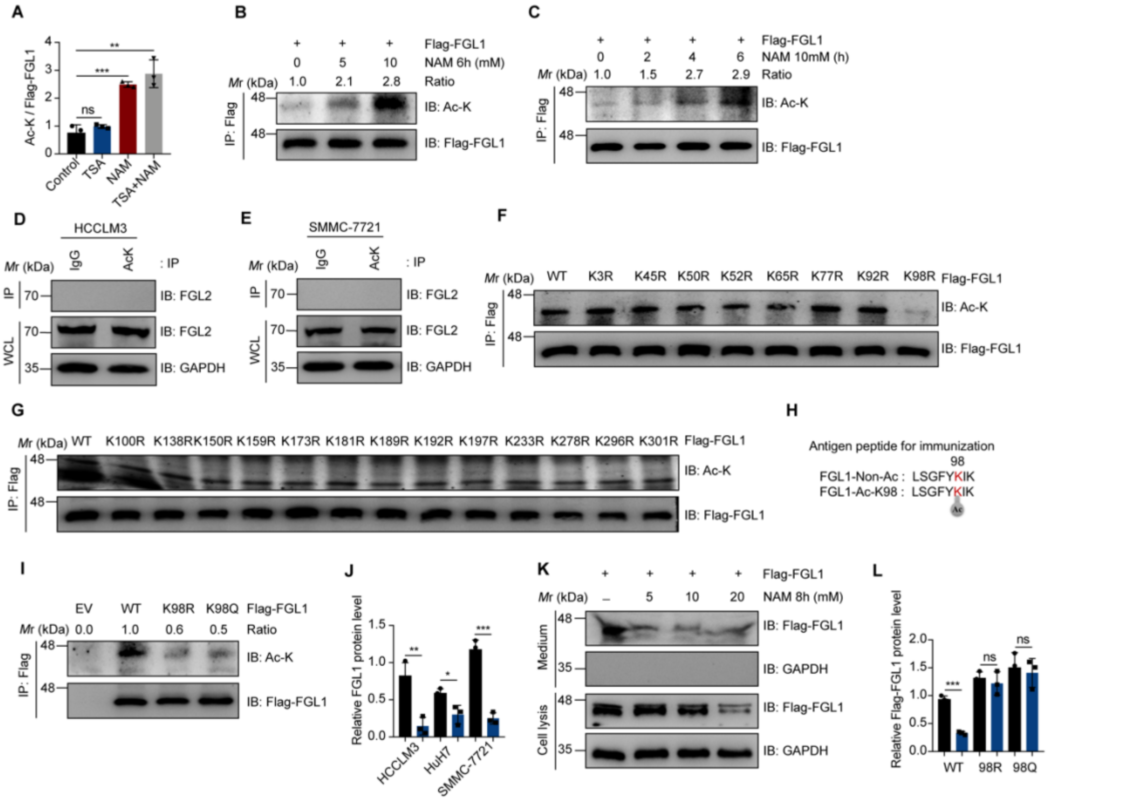

Supplemental Figure 1. FGL1 is acetylated at Lys 98 and acetylation promotes its degradation.

(**A**) Statistical analysis of immunoblots in Figure 1C determined by Student's t test. ns, no significance, * $p < 0.05$, ** $p < 0.01$, *** $p < 0.001$. (**B**) IB analysis of FGL1 acetylation level in HEK293T cells stably expressed Flag-FGL1 and treated with increasing concentrations of NAM (0 to 10 mM) for 6h. (**C**) IB analysis of FGL1 acetylation level in HEK293T cells stably expressed Flag-FGL1 and treated with 10 mM NAM for different time as indicated. (**D**) IB analysis of whole-cell lysates (WCL) and anti-acetylated lysine immunoprecipitates (IPs) derived from HCCLM3 cells. IgG was used as a negative control. (**E**) IB analysis of whole-cell lysates (WCL) and anti-acetylated lysine immunoprecipitates (IPs) derived from SMMC-7721 cells. IgG was used as a negative control. (**F**) IB analysis of FGL1acetylation derived from HEK293T cells transfected with Flag-FGL1 WT or different mutant forms. (**G**) IB analysis of FGL1acetylation derived from HEK293T cells transfected with Flag-FGL1 WT or different mutant forms. (**H**) A schematic diagram of the FGL1 Lys 98 non-acetylated and acetylated peptides used for immunization

to generate the anti-acetyl-K98 FGL1 antibody. (**I**) K98R and K98Q mutations decrease FGL1 acetylation. (**J**) Statistical analysis of immunoblots in Figure 1H determined by Student's t test. ns, no significance, * $p < 0.05$, ** $p < 0.01$, *** $p < 0.001$. (**K**) IB analysis of FGL1 protein level in HEK293T cells stably expressed Flag-FGL1 and treated with increasing concentrations of NAM (0 to 10 mM) for 8h. (**L**) Statistical analysis of immunoblots in Figure 1K determined by Student's t test. ns, no significance, * $p < 0.05$, ** $p < 0.01$, *** $p < 0.001$.

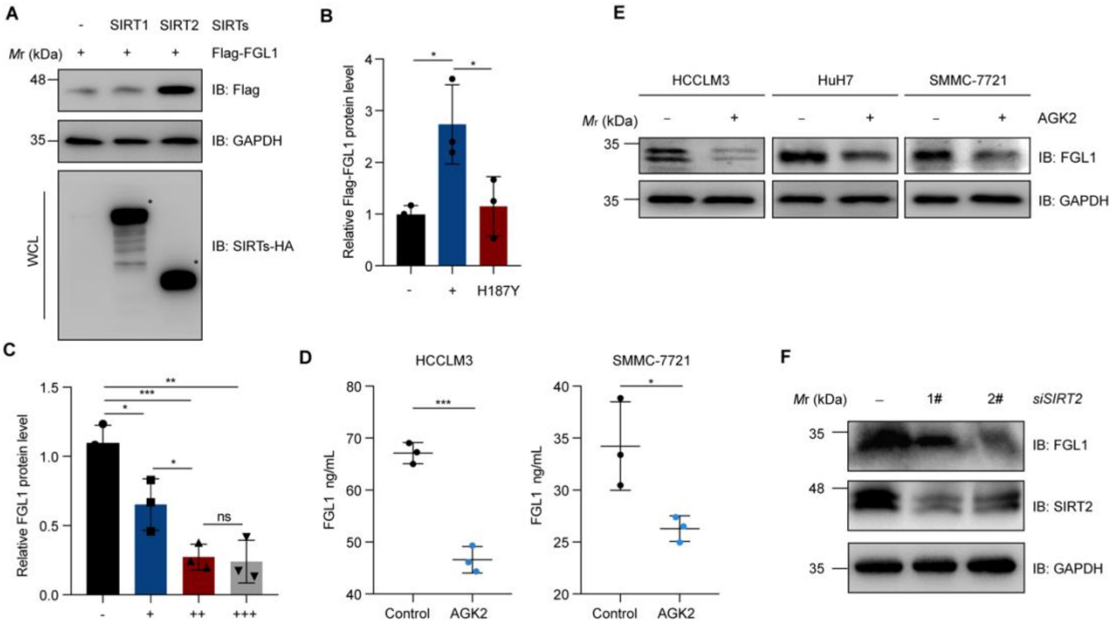

Supplemental Figure 2. Inhibition of SIRT2 reduces FGL1 protein level. (**A**) IB analysis of FGL1 level in HEK293T cells stably expressed Flag-FGL1 and transfected with SIRT1 or SIRT2. (**B**) Statistical analysis of immunoblots in Figure 2I determined by Student's t test. ns, no significance, * $p < 0.05$, ** $p < 0.01$, *** $p < 0.001$. (**C**) Statistical analysis of immunoblots in Figure 2K determined by Student's t test. ns, no significance, * $p < 0.05$, ** $p < 0.01$, *** $p < 0.001$. (**D**) The amounts of soluble FGL1 from different HCC cell lines treated with or without AGK2 (20 μM,8h) were assessed by Enzyme-linked immunoassay (ELISA) ($n = 3$) with Student's t test. ns, no significance, * $p < 0.05$, ** $p < 0.01$, *** $p < 0.001$. (**E**) IB analysis of endogenous FGL1 level in multiple HCC cell lines treated with AGK2 (20 μM,8h). (**F**) IB analysis of endogenous FGL1 level in control and *SIRT2* knockdown HCCM3 cells.

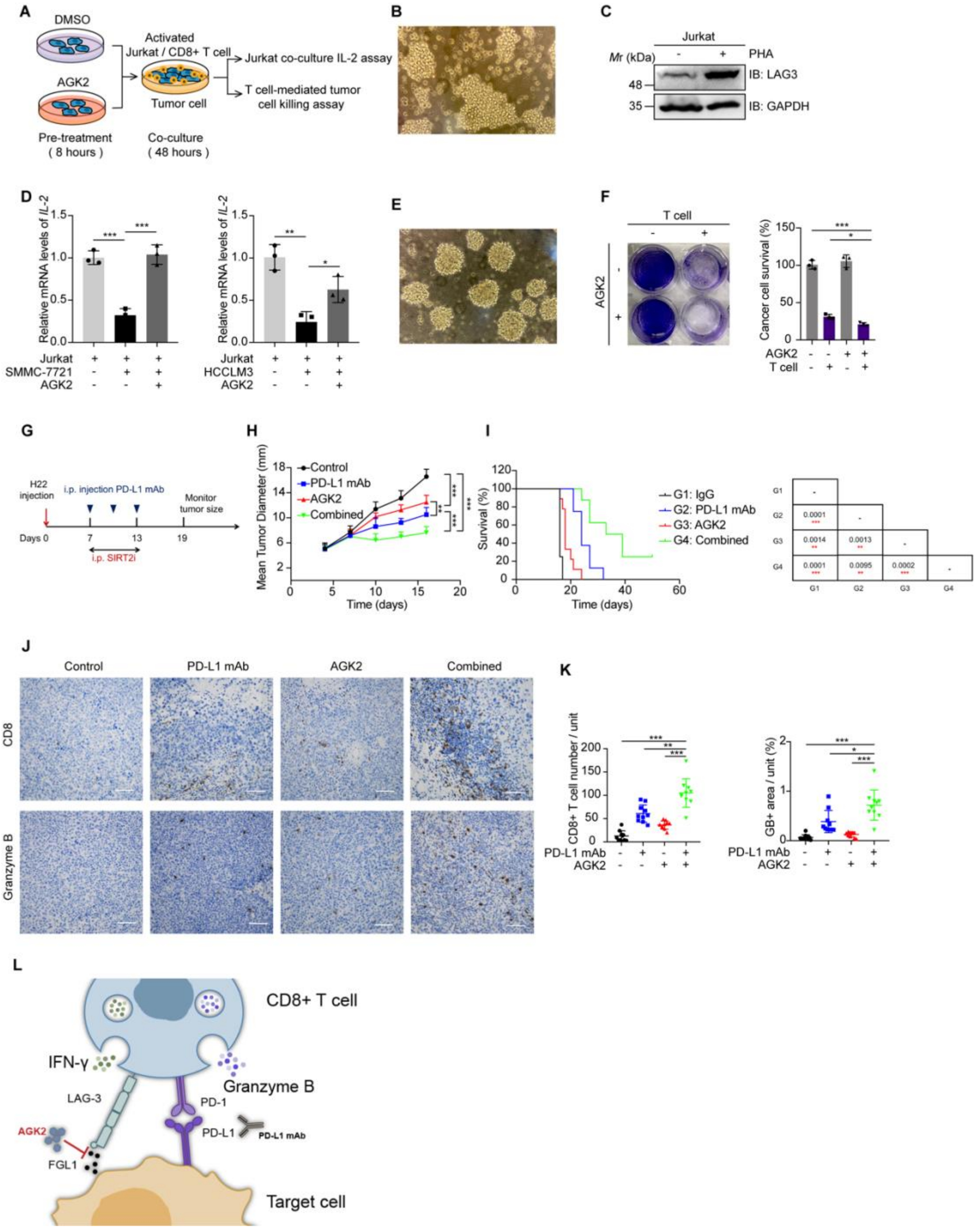

Supplemental Figure 3. The combination of AGK2 and PD-L1 blockade effectively suppresses tumor growth of HCC. (**A**) Schematic representation of established in vitro co-culture system. (**B**) Morphology of activated Jurkat T cells. (**C**) IB analysis of LAG3 level in Jurkat T cells stimulated with or without PHA (500 ng/mL, 48 h). (**D**) qPCR analysis of *IL-2* mRNA levels in Jurkat T cells co-cultured with or without AGK2 pretreated HCCLM3 or SMMC-7721 cells. Data are mean ± S.D. of n = 3

independent experiments. Statistical differences were determined by *t*-test. * $p < 0.05$, ** $p < 0.01$, *** $p < 0.001$. (**E**) Morphology of activated human T cells. (**F**) Images of T cell-mediated cancer cell killing assay. HCCLM3 cells pretreated with AGK2 were co-cultured with activated T cell for 48 h. Crystal violet staining was used for quantification. Data are mean ± S.D. of n = 3 independent experiments. (**G**) Schematic representation of the experimental procedure. (**H**) Tumor growth of H22 murine hepatocarcinoma tumor cells in BALB/c mice treated with control (black lines; n=8), AGK2 (red lines; n=9), anti-PD-L1 mAb (blue lines; n=8) or the combined therapy (green lines; n=8). Tumor growth is shown as mean tumor diameter ± S.D. (**I**) Kaplan–Meier survival curves for each treatment group (control, n= 8; PD-L1 mAb, n= 8; AGK2, n= 9; combined, n= 8). The *p* value was calculated using a two-sided Gehan–Breslow–Wilcoxon test. (**J**) Immunohistochemistry of $CD8^+$ T cell infiltration and granzyme B in the H22 tumor mass as indicated. Scale bars, 100 μm. Data represent mean ± S.D. from 5 independent samples of each group. (**K**) IHC results of (J) were quantified and the statistical differences were determined by Student's t test. ns, no significance, * $p < 0.05$, ** $p < 0.01$, *** $p < 0.001$. (**L**) Schematic diagram showing how AGK2 enhances the immunotherapy response through decreasing FGL1 protein levels in HCC cell lines and blocking LAG-3/FGL1 pathway.

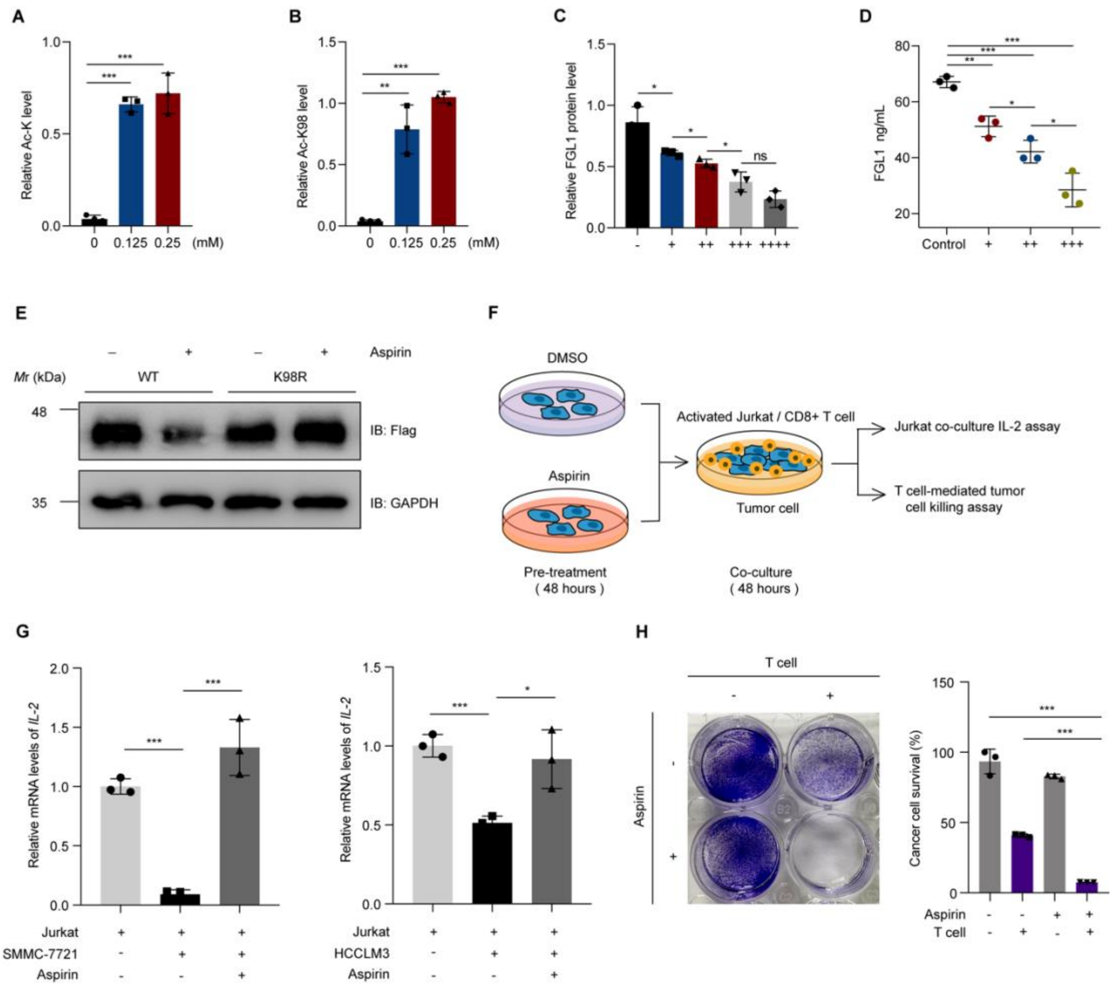

Supplemental Figure 4. Aspirin acetylates FGL1 and promotes its degradation. (**A**) Statistical analysis of immunoblots in Figure 4A determined by Student's t test. ns, no significance, * $p < 0.05$, ** $p < 0.01$, *** $p < 0.001$. (**B**) Statistical analysis of immunoblots in Figure 4B determined by Student's t test. ns, no significance, * $p < 0.05$, ** $p < 0.01$, *** $p < 0.001$. (**C**) Statistical analysis of immunoblots in Figure 4G determined by Student's t test. ns, no significance, * $p < 0.05$, ** $p < 0.01$, *** $p < 0.001$. (**D**) Enzyme-linked immunoassay (ELISA) quantification of soluble FGL1 from HCCLM3 when treated with or without aspirin (+, 0.125mM; ++, 0.25mM; +++, 0.5mM) for 24h. Statistical differences were determined by t-test. * $p < 0.05$, *** $p < 0.001$. 3 biological replicates were analyzed. (**E**) IB analysis of FGL1 protein level in HEK293T cells transfected with FGL1 WT or 98R mutant under treatment of aspirin (0.5mM, 24h). (**F**) Schematic representation of established in vitro co-culture system. (**G**) qPCR analysis

of *IL-2* mRNA level in stimulated Jurkat T cells co-cultured with control or aspirin pretreated HCCLM3 or SMMC-7721 cells. Data are mean ± S.D. of n = 3 independent experiments. Statistical differences were determined by t-test. * $p < 0.05$, *** $p < 0.001$. (**H**) Images of T cell-mediated cancer cell killing assay. HCCLM3 cells pretreated with aspirin were then co-cultured with activated T cell for 48 h. Crystal violet staining was used for quantification. Data are mean ± S.D. of n = 3 independent experiments.

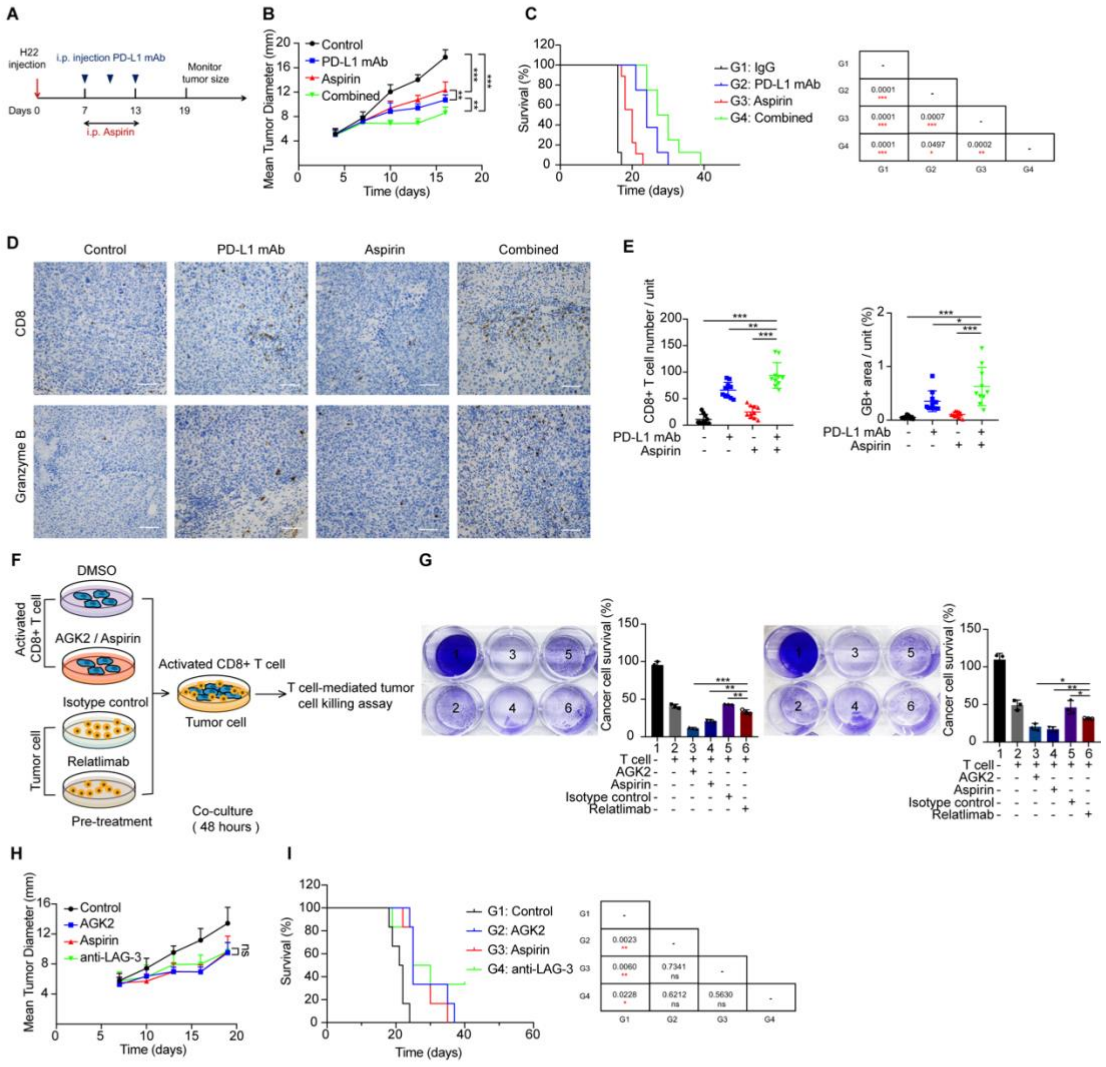

Supplemental Figure 5. Aspirin downregulates FGL1 and enhances the efficacy of anti-PD-L1 immunotherapy. (**A**) Schematic representation of the experimental procedure. (**B**) Tumor growth of H22 murine hepatocarcinoma tumor cells in BALB/c mice treated with control (black lines; n=8), aspirin (red lines; n=9), anti-PD-L1 mAb (blue lines; n=8) or the combined therapy (green lines; n=8). Tumor growth is shown as mean tumor diameter ± S.D. (**C**) Kaplan–Meier survival curves for each treatment group (control, n= 8; PD-L1 mAb, n= 8; aspirin, n= 9; combined therapy, n= 8). The *p* value was calculated using a two-sided Gehan–Breslow–Wilcoxon test. (**D**) Immunohistochemistry of CD8^{+} T cell infiltration and granzyme B in the H22 tumor mass as indicated. Scale bars, 100 μm. Data represent mean ± S.D. from 5 independent samples of each group. (**E**) IHC results of (E) were quantified and the statistical

differences were determined by Student's t test. ns, no significance, * $p < 0.05$, ** $p < 0.01$, *** $p < 0.001$. (**F**) Schematic representation of established in vitro co-culture system. (**G**) Images of T cell-mediated cancer cell killing assay. HCCLM3 and SMMC-7721 cell lines pretreated with AGK2 or aspirin were co-cultured with activated T cell for 48 h. Activated T cell pretreated with Relatlimab were co-cultured with HCCLM3 and SMMC-7721 cell lines for 48 h. Crystal violet staining was used for quantification. Data are mean ± S.D. of n = 3 independent experiments. Statistical differences were determined by Student's t test. ns, no significance, * $p < 0.05$, ** $p < 0.01$, *** $p < 0.001$. (**H**) Tumor growth of Hepa 1-6 cells in C57BL/6 mice treated with control (black lines), aspirin (red lines), AGK2 (blue lines) or the anti-LAG-3 mAb (green lines). n = 6 biologically independent animals per group. Tumor growth is shown as mean tumor diameter ± S.D. (**I**) Kaplan–Meier survival curves for each treatment group. n = 6 biologically independent animals per group. The *p* value was calculated using a two-sided Gehan–Breslow–Wilcoxon test.

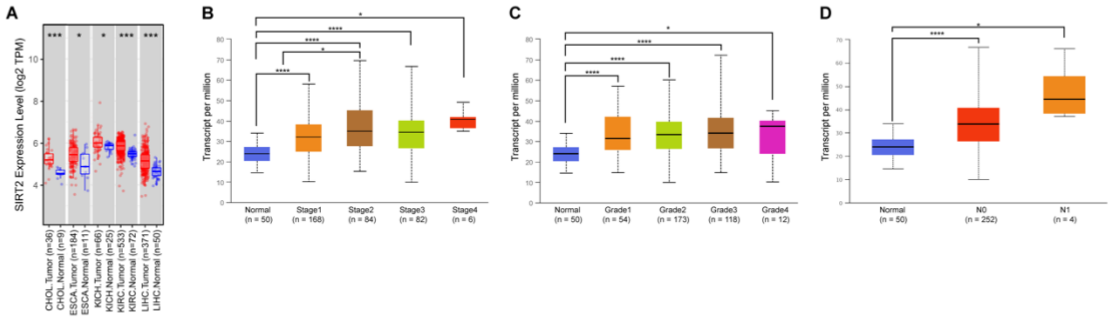

Supplemental Figure 6. SIRT2 is upregulated while K98-Acetylation of FGL1 is downregulated in hepatocellular carcinoma. (**A**) The mRNA levels of SIRT2 derived from normal or tumor specimen in various cancer types from TCGA database. Statistical differences were determined by Student's t test. CHOL: Cholangiocarcinoma; ESCA: Esophageal carcinoma; KICH: Kidney Chromophobe; KIRC: Kidney renal clear cell carcinoma; LIHC: Liver hepatocellular carcinoma. (**B-D**) SIRT2 expression analysis in HCC based on tumor stages, grades and nodal metastasis status by UALCAN.

Supplementary Tables

Supplemental Table 1.

qPCR primers		
	**Forward 5'-3'**	**Reverse 5'-3'**
**hACTIN**	TATTGGCAACGAGCGGTTCC	GGCATAGAGGTCTTTACGGATGTC
**hGAPDH**	GTCTCCTCTGACTTCAACAGCG	ACCACCCTGTTGCTGTAGCCAA
**hIL-2**	GAATGGAATTAATAATTACAAGAATCCC	TGTTTCAGATCCCTTTAGTTCCAG
**hFGL1 1-1**	ATGGCAAAGGTGTTCAGTTTCA	ACAATCTGCATACTGCCTCTTG
**hFGL1 1-2**	TATTGTGACATGTCCGATGGAGG	TTCAGAGAATACCACCACCCATG
**hFGL1 1-3**	CATTGATCTTGGAAGCAAGAGGC	AGTTGTCATGATCTCTGTCCCAC

Supplemental Table 2.

Antibodies used for immunoblot		
**Antibody**	**Source**	**Cat Number**
**FGL1**	Proteintech	16000-1-AP
**FGL1**	Abcam	ab170922
**FGL2**	Proteintech	67152-1-Ig
**GAPDH**	Proteintech	60004-1-Ig
**Acetylated-lysine antibody**	Cell Signaling Technology	#9441
**LAG3**	Proteintech	16616-1-AP
**His-tag**	HUABIO	R1207-2
**HA-tag**	AbHO	HOA012HA01
**Flag-tag**	AbHO	HOA012FL01
**Secondary anti-mouse**	SAB	L3032
**Secondary anti-rabbit**	SAB	L3012
**InVivoMAb rat IgG2a isotype control**	BioXcell	BE0089
**InVivoMab anti-mouse PD-L1 (B7-H1)**	BioXcell	BE0101
**InVivoMAb rat IgG1 isotype control**	BioXcell	BE0088
**InVivoMAb anti-mouse LAG-3**	BioXcell	BE0174

Supplemental Table 3.

Antibodies used for IHC staining		
**Antibody**	**Source**	**Cat Number**
**CD8α**	Cell Signaling Technology	98941
**Granzyme B**	Abcam	ab255598
**FGL1**	Abcam	ab275091
**SIRT2**	Abcam	ab211033
**Acetyl-K98-FGL1**	ABclonal Technology Biotech	N/A

Supplemental Table 4.

**The correlation of clinicopathological variables of the HCC patients and SIRT2 expression levels**					
**Clinicopathological parameters**	**n**	**SIRT2 expression**		**χ^2**	**P value**
		**Low**	**High**		
**all**	84	47	37		
**Age(year)**				1.097	0.295
<55	44	27	17		
≥55	40	20	20		
**Gender**				1.948	0.163
Male	75	40	35		
Female	9	7	2		
**Liver cirrhosis**				0.398	0.528
No	49	26	23		
Yes	35	21	14		
**Tumor size(cm)**				12.755	0.000*
≤5	34	27	7		
>5	50	20	30		
**TNM stage**				15.452	0.000*
I/II	43	33	10		
III/IV	41	14	27		
**Pathological grade**				3.657	0.454
I/I-II	10	8	2		
II/II-III	68	37	31		
III/III-IV	6	2	4		

[a] A chi-square test was used for comparing groups between low and high SIRT2 expression. *, p<0.05 was considered significant.

Supplemental Table 5.

**The correlation of clinicopathological variables of the HCC patients and FGL1 expression levels**					
**Clinicopathological parameters**	**n**	**FGL1 expression**		χ^2	**P value**
		**Low**	**High**		
**all**	84	35	49		
**Age(year)**				1.859	0.173
<55	44	19	25		
≥55	40	16	24		
**Gender**				2.592	0.107
Male	75	29	46		
Female	9	6	3		
**Liver cirrhosis**				3.931	0.047*
No	49	16	33		
Yes	35	19	16		
**Tumor size(cm)**				9.492	0.002*
≤5	34	21	13		
>5	50	14	36		
**TNM stage**				7.254	0.007*
I/II	43	24	19		
III/IV	41	11	30		
**Pathological grade**				4.727	0.316
I/I-II	10	4	6		
II/II-III	68	31	37		
III/III-IV	6	0	6		

[a] A chi-square test was used for comparing groups between low and high SIRT2 expression. *, $p<0.05$ was considered significant.

Supplemental Table 6.

The correlation of clinicopathological variables of the HCC patients and Ac-K98 / total FGL1 expression levels					
**Clinicopathological parameters**	**n**	**Ac-K98 / total FGL1**		**χ^2**	**P value**
		**Low**	**High**		
**all**	84	37	47		
**Age(year)**				0.028	0.867
<55	44	19	25		
≥55	40	18	22		
**Gender**				1.948	0.163
Male	75	35	40		
Female	9	2	7		
**Liver cirrhosis**				1.160	0.281
No	49	24	25		
Yes	35	13	22		
**Tumor size(cm)**				9.757	0.002*
≤5	34	8	26		
>5	50	29	21		
**TNM stage**				12.189	0.000*
I/II	43	11	32		
III/IV	41	26	15		
**Pathological grade**				6.103	0.047*
I/I-II	10	2	8		
II/II-III	68	30	38		
III/III-IV	6	5	1		

[a] A chi-square test was used for comparing groups between low and high SIRT2 expression. *, $p<0.05$ was considered significant.

Supplemental Table 7.

**The analysis of prognosis makers of HCC**							
**Variables**	**n**	**Univariate analyses**			**Multivariate analyses**		
		**HR**	**(95% CI)**	**P value**	**HR**	**(95% CI)**	**P value**
**Age(year)**				0.627			0.329
<55	44						
≥55	40	0.873	0.505-1.508		0.739	0.403-1.355	
**Gender**				0.360			0.779
Male	75						
Female	9	1.611	0.579-4.476		1.176	0.377-3.661	
**Liver cirrhosis**							0.046*
No	49			0.613			
Yes	35	1.152	0.665-1.993		1.802	1.011-3.211	
**Tumor size(cm)**				0.015*			0.236
≤5	34						
>5	50	2.088	1.151-3.786		0.499	0.158-1.573	
**TNM stage**				0.000*			0.060
I/II	43						
III/IV	41	3.068	1.706-5.517		2.755	0.957-7.930	
**Pathological grade**				0.051			0.333
I/I-II	10						
II/II-III	68						
III/III-IV	6	1.933	0.997-3.746		1.422	0.696-2.904	
**SIRT2**				0.000*			0.014*
Low	47						
High	37	5.194	2.824-9.550		2.642	1.215-5.742	
**FGL1**				0.000*			0.045*
Low	35						
High	49	3.688	1.935-7.026		2.187	1.018-4.699	
**Ac-K98 / total FGL1**				0.000*			0.006*
Low	37						
High	47	0.182	0.099-0.333		0.366	0.179-0.750	

Abbreviations: 95% CI, 95% confidence interval; HR, hazard ratio. *****, p<0.05 was regarded as statistically significant, p value was calculated using Cox's proportional hazards regression.
